# Dual-purpose zinc and silicon complexes of 1,2,3-triazole group substituted phthalocyanine photosensitizers: synthesis and evaluation of photophysical, singlet oxygen generation, electrochemical and photovoltaic properties†

**DOI:** 10.1039/c8ra10665g

**Published:** 2019-04-08

**Authors:** Emre Güzel

**Affiliations:** Department of Chemistry, Sakarya University TR54050 Serdivan Sakarya Turkey emreguzel_@outlook.com eguzel@sakarya.edu.tr +90-264-295-74-26

## Abstract

The synthesis, photophysical, singlet oxygen generation, electrochemical and photovoltaic properties of peripheral and axial 1,2,3-triazole group substituted zinc and silicon phthalocyanine complexes with strong absorption in the visible region were described. All novel complexes have been characterized by spectroscopic and electrochemical techniques. All the new compounds are highly soluble in most common organic solvents. The electronic absorption and fluorescence spectral properties of complexes 4 and 5 are investigated. The effects of the triazole group, different metal centers and position of the substituent on the photophysical, electrochemical and photovoltaic properties of the new phthalocyanines were also investigated for the first time in this work. According to the fluorescence measurements, the axially substituted silicon complex (5) showed higher fluorescence quantum yield (*Φ*_F_ = 0.28) than the peripherally substituted zinc complex (4). In addition, quantum yields for singlet oxygen generation (*Φ*_Δ_ = 0.32 for silicon complex (4) and *Φ*_Δ_ = 0.76 for zinc complex (5) in DMSO) were obtained. Electrochemical studies show that complex 5 is present in non-aggregated form as a result of steric hindrance of the axial groups; the LUMO level of this complex is slightly more negative than the conduction band of TiO_2_ and electron injection might be less effective. Therefore, the power conversion efficiency of 1.30% for a complex 4 based dye-sensitized solar cell (DSSC) is higher than complex 5 (0.90%). Consequently, these zinc and silicon complexes are promising candidates not only for photodynamic therapy but also solar power conversion.

## Abstract 2

Evaluation of dual-purpose zinc and silicon phthalocyanine complexes on photophysical, singlet oxygen generation, electrochemical and photovoltaic properties.

## Graphical abstract

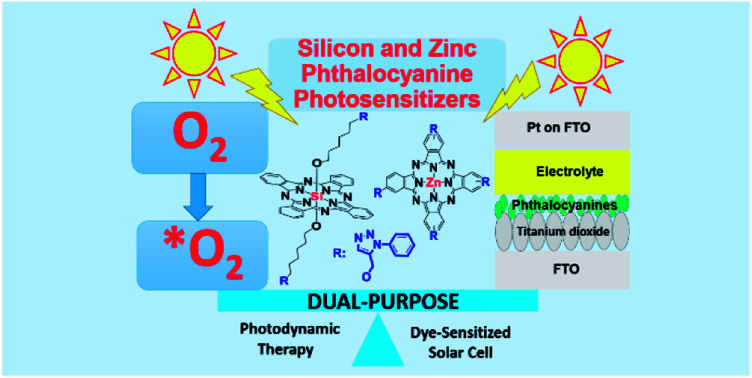

## 1. Introduction

Triazole groups, especially 1,2,4-triazoles and 1,2,3-triazoles, are important nitrogen heterocycles that have been widely studied as essential structural features of many of the potent azole fungicides.^1^ In recent years, chemical substances containing the 1,2,3-triazole group have been used as light stabilizers and optical brighteners. 1,2,3-Triazole moieties may be advantageous in binding biological molecular targets as they are stable against metabolic degradation, are capable of hydrogen bonding, and may increase the solubility of the compound.^2,3^ Although 1,2,3-triazole groups do not occur naturally, synthetic molecules containing 1,2,3-triazole units exhibit a variety of biological activities, and they have been utilized as starting compounds for the synthesis of many heterocycles. The importance of triazolic compounds in medicinal chemistry is undeniable. Triazole derivatives are also produced as a drug under varying different trade names such as anastrozole and letrozole for cancer and fluconazole, albaconazole, voriconazole and isavuconazole for anti-fungal treatment.^4^ In addition, derivatives containing the 1,2,3-triazole group are used as antinuclear agents, HIV-1 protease inhibitors, human β3-adrenergic receptors, and antituberculosis, antibacterial, miscellaneous, and anticancer agents.^5^ It is also known that pyridine derivatives form a coordination bond with the TiO_2_ surface.^6^ Therefore, the 1,2,3-triazole groups can be used as anchoring groups such as pyridine derivatives for dye-sensitized solar cells (DSSCs) due to these heterocyclic ligands.

Phthalocyanines (Pcs) have high chemical and thermal stability, and high molar absorption coefficients and exhibit rich and diverse chemistry as well as specific optical, electronic, structural, and coordination properties. There has been great interest in phthalocyanines for use in photodynamic cancer therapy, especially due to their high absorption coefficient where penetration of light into tissue is optimal. The ability to generate reactive oxygen species, as well as these features that mainly produce singlet oxygen in the photodynamic process, have made suitable candidates for photodynamic therapy. Singlet oxygen is considered to be the primary cytotoxic agent that plays an important role in the photodynamic therapy of cancer. Single oxygen production requires the irradiation of an appropriate wavelength for stimulation of an effective photosensitizer, dissolved molecular oxygen, and a photosensitizer. A promising photosensitizer should have the ability to perform an efficient intersection transition so that it can transfer energy to underground-state molecular oxygen.^7,8^ Phthalocyanines are the leading photosensitizers with this property. The first step in PDT treatments is to synthesize suitable photosensitizers and to evaluate the photophysical and photochemical properties of this photosensitizers and to test its usability for the PDT. It is a fact that the metal atom coordinated to the phthalocyanine ring can alter the physical and chemical properties of the complex.^9^ Phthalocyanines with these properties are widely used as photosensitizers in both photodynamic cancer treatment and dye-sensitized solar cell (DSSC) applications.^9–12^ Particularly zinc and silicon metal coordinated phthalocyanine complexes have been used in the photodynamic therapy of cancer owing to the closed shell, diamagnetic ion (such as Si^4+^, Zn^2+^) give both high triplet quantum yields and long lifetimes.^13–16^ Also due to this diamagnetic metal properties, zinc phthalocyanines that have been studied widely by many groups are very important photosensitizers for DSSC applications. In addition, Zn(ii) phthalocyanine complexes are used as photosensitizers owing to their long-lived (>1 ns) singlet excited states. Metal-free porphyrins and phthalocyanines are generally more ineffective photosensitizers than Zn(ii) analogs and show a significant decrease in photo-current response due to low excited singlet states (the oxidation potential of free bases are generally more positive whereas the HOMO–LUMO gaps stay similar).^17^

Chemical structure of substituents which are attached to the peripheral or non-peripheral positions of phthalocyanines significantly change their properties such as absorption behavior and solubility.^9,18–23^ According to the positions of the substituents, it is readily understood that two different tetra-substituted phthalocyanines differ significantly in their photophysical and photochemical behaviors. Similarly, metal atoms coordinated to the phthalocyanine center are known to alter the electrochemical properties of the compound. The applications of the metallophthalocyanine complexes are almost entirely based on electron transfer reactions due to the 18 π-electron-conjugated system. In many applications of phthalocyanines, it is important to have a good understanding of the redox properties of these compounds.^24–26^

Taking these properties into consideration, it has focused on non-aggregate complexes as a strategy to explain Pcs in conjunction with the 1,2,3-triazole group to study aggregation, photophysical, singlet oxygen generation, electrochemical and photovoltaic properties. Besides the electrochemical behavior of MPcs was investigated so as to support the proposed structure. Determination of the electrochemical behavior of complexes will be important in assessing the possible use of complexes in different electrochemical fields such as electrocatalysis and electrosensing. In the literature, there are only few studies about the synthesis of 1,2,3-triazole-substituted phthalocyanines,^27^ despite there are many important studies about 1,2,4-triazole-substituted phthalocyanines.^28–31^ In addition, photo-physicochemical and electrochemical properties of phthalocyanines have widely known but the examination of these properties have been made for only few of 1,2,3-triazole bearing phthalocyanines. There are no studies examining and comparing all the photophysical, singlet oxygen generation, electrochemical and photovoltaic properties of peripheral substituted zinc and axial substituted silicon phthalocyanine complexes. Also, to the best of our knowledge, there is no report on the utilization of 1,2,3-triazole unit as an anchoring group into a dye molecule for DSSCs. This is the first study using the 1,2,3-triazole group as an anchoring group in DSSCs. Therefore, 1,2,3-triazole-substituted silicon and zinc phthalocyanines were designed and prepared. Also, their photo-physicochemical, electrochemical and photovoltaic properties were investigated.

## 2. Experimental

The used equipment, materials, and the photophysical, photochemical, electrochemical and photovoltaic parameters were supplied as ESI.†

### 2.1. Synthesis

#### 2.1.1. 6-((1-Phenyl-1*H*-1,2,3-triazol-5-yl)methoxy)hexan-1-ol (2)

(1-Phenyl-1*H*-1,2,3-triazol-5-yl)methanol (3.5 g, 0.02 mol) dissolved in ethanol (25 mL), KOH (1.44 g, 0.025 mol) was added in it by stirring at 45 °C for 1 h and then, 6-chlorohexan-1-ol (2.72 g, 0.02 mol) in ethanol (5 mL) was added drop by drop to the first solution while stirring efficiently for 1 h. After this product was refluxed for 48 h under a nitrogen atmosphere and then reaction cooled. The solvent in the reaction medium was removed by vacuum and the obtained crude product was dissolved in CHCI_3_ (80 mL) and cleaned three times with 10% NaOH (2.15 mL) and three times with water (100 mL). The organic phase was dried with MgSO_4_, filtered and evaporated. The crude product was purified with an aluminum oxide column using CHCI_3_ as a solvent. The oily yellow product was dried *in vacuo* (P_2_O_5_). This product was soluble in THF, CHCl_3_, CH_2_Cl_2_, DMF, and DMSO. Yield: 3.3 g (60%). FT-IR (*ν*_max_/cm^−1^): 3372 (–O–H), 3103 (Ar–C–H), 2934–2861 (Aliph. –C–H), 1599 (Ar–C=C), 1503, 1447 (–N=N triazole), 1352 (–C–N triazole) 1232, 1109, 1044, 759, 691, 522. ^1^H-NMR (300 MHz, CDCl_3_): *δ* 7.99 (s, 1H), 7.69 (m, 2H), 7.47 (m, 3H), 4.83 (s, 2H), 3.49 (m, 4H), 1.30 (m, 8H). ^13^C-NMR (75 MHz, CDCl_3_): *δ* 137.16, 129.99 (overlapped 2C signals), 129.06, 120.77 (overlapped 2C signals), 120.39, 70.86, 64.46, 62.98, 32.84, 29.79, 26.13, 25.76. Anal. calc. for C_15_H_21_N_3_O_2_: C, 65.43; H, 7.69; N, 15.26; O, 11.62; found: C, 65.13; H, 7.39; N, 15.46; O, 11.62. MS MALDI-TOF: *m*/*z* 275.82 [M]^+^.

#### 2.1.2. 4-((1-Phenyl-1*H*-1,2,3-triazol-5-yl)methoxy)phthalonitrile (3)

4-Nitrophthalonitrile (2.0 g, 11.2 mmol) and (1-phenyl-1*H*-1,2,3-triazol-5-yl)methanol (1.96 g, 11.6 mmol) were dissolved in 40 mL DMF at 45 °C under argon atmosphere. To the reaction mixture was added 5 portions of potassium carbonate (9 g, 65.2 mmol) every 8 hours. After stirring for 48 hours, the mixture was slowly cooled to room temperature. It was then poured into 250 mL of ice–water mixture. After filtration, the crude product was purified by using column chromatography on silica gel (methanol/chloroform 1/50). Yellowish-white product was soluble in ethylacetate, THF, CHCl_3_, CH_2_Cl_2_, and DMF. Yield: 1.38 g, (42%). FT-IR (*ν*_max_/cm^−1^): 3078 (Ar–C–H), 2984 (Aliph. –C–H), 2230 (–C≡N), 1602 (Ar–C=C), 1564, 1490, 1405 (–N=N triazole), 1309 (–C–N triazole), 1254 (R–O–Ar), 1008, 759, 685, 526. ^1^H-NMR (300 MHz, CDCl_3_): *δ* 8.11 (s, 1H), 7.75 (m, 3H), 7.48 (m, 5H), 5.40 (s, 2H). ^13^C-NMR (75 MHz, CDCl3): *δ* 161.38, 142.80, 136.88, 135.61, 130.14 (overlapped 2C signals), 129.51, 121.88, 120.87 (overlapped 2C signals), 120.55, 119.74, 117.72, 115.78, 115.37, 108.16, 62.74. Anal. calc. for C_17_H_11_N_5_O: C, 67.77; H, 3.68; N, 23.24; O, 5.31; found: C, 66.04; H, 3.33; N, 23.66; O, 5.55. MS MALDI-TOF: *m*/*z* 301.43 [M]^+^.

#### 2.1.3. Zinc(ii) phthalocyanine (4)

A mixture of 4-((1-phenyl-1*H*-1,2,3-triazol-5-yl)methoxy)phthalonitrile (0.100 g, 0.332 mmol), anhydrous Zn(CH_3_COO)_2_ (0.041 g, 0.234 mmol) and DBU (0.25 mmol) in amyl alcohol (2 mL) was refluxed at 140 °C for 8 hours. The mixture was cooled to room temperature and precipitated by addition of *n*-hexane. The product was washed with hot ethanol, acetone and ethyl acetate in order to remove the impurities. The targeted pure product was obtained by basic silica gel column chromatography using a gradient of chloroform/ethanol (9/1). Yield: 0.036 g, (35%). FT-IR (*ν*_max_/cm^−1^): 3102 (Ar–C–H), 2958–2871 (Aliph. –C–H), 1608 (Ar–C=C), 1487, 1405, 1337 (–C–N triazole), 1232 (R–O–Ar), 1038, 948, 819, 744, 643, 470. UV-Vis *λ*_max_ (nm) THF: 676, 610, 348. ^1^H-NMR (300 MHz, CDCl_3_): *δ*, ppm 9.18 (s, 4H, Triazole Ar–H), 8.84–7.46 (32H, m, Pc–Ar–H, and Ar–H), 5.75 (8H, s, OCH_2_). Anal. calc. for C_68_H_44_N_20_O_4_Zn: C, 64.28; H, 3.49; N, 22.05; O, 5.04; Zn, 5.15; found: C, 64.04; H, 3.33; N, 22.66. MS MALDI-TOF: *m*/*z* 1270.50 [M]^+^.

#### 2.1.4. Silicon(iv) phthalocyanine (5)

A mixture of unsubstituted dichloro[phthalocyaninato]silicon (50 mg, 0.0817 mmol), NaH (0.735 mmol, 17.5 mg) and 6-((1-phenyl-1*H*-1,2,3-triazol-5-yl)methoxy)hexan-1-ol (66.21 mg, 0.244 mmol) in dry toluene (20 mL) was refluxed for 8 h under N_2_ atmosphere. The reaction mixture was centrifuged, the filtrate removed and the residue washed with *n*-hexane (2 × 40 mL) and dried *in vacuo*. The obtained crude product was purified by aluminum oxide column chromatography and using dichloromethane/methanol (20/1) as eluent. Yield: 57 mg (64.1%). FT-IR (*ν*_max_/cm^−1^): 3081 (Ar–C–H), 2929–2849 (Aliph. –C–H), 1601 (Ar–C=C), 1519, 1433, 1428 (–N=N triazole), 1332 (–C–N triazole), 1291, 1024, 909, 736, 645, 573, 530. UV-Vis *λ*_max_ (nm) THF: 671, 605, 354. ^1^H-NMR (300 MHz, CDCl_3_): *δ*, ppm 9.56 (s, 8H, Pc–H_α_), 8.26 (s, 8H, Pc–H_β_), 7.92–7.26 (m, 2H, triazole Ar–H and m, 10H, benzene Ar–H), 4.61–(−1.76) (m, 28H, CH_2_–O and Aliph. CH). Anal. calc. for C_62_H_56_N_14_O_4_Si: C, 68.36; H, 5.18; N, 18.00; O, 5.87; Si, 2.58; found: C, 68.04; H, 5.33; N, 18.66. MS MALDI-TOF: *m*/*z* 1095.95 [M + 6H]^+^.

## 3. Results and discussion

### 3.1. Synthesis and characterization

**Scheme 1:**
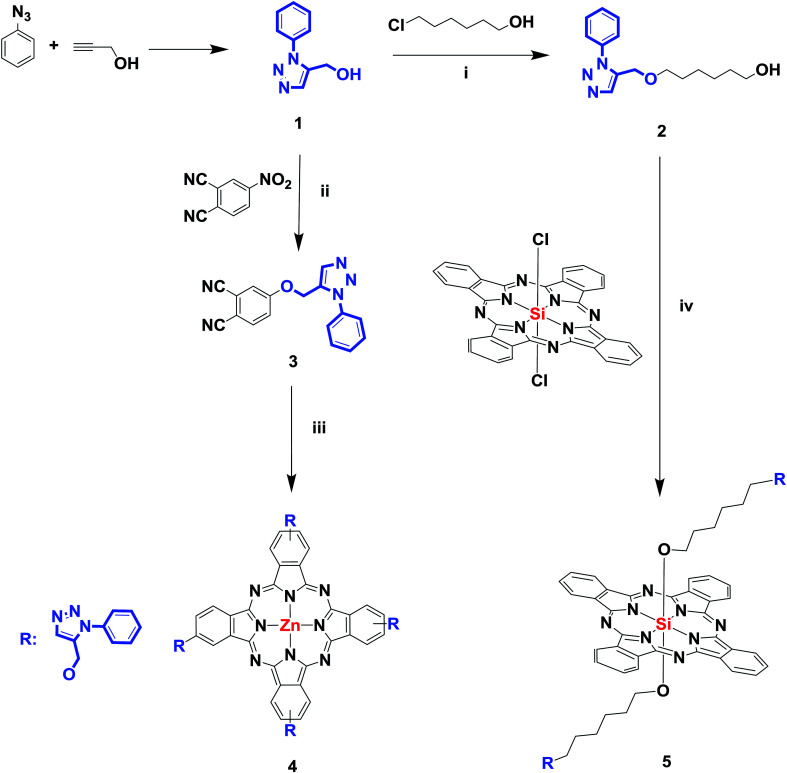
The synthesis of compound 1, 2, phthalonitrile derivative 3 and it's zinc and silicon complexes (4 and 5) (i) NaOH, EtOH, 80 °C. (ii) K_2_CO_3_, DMF, 45 °C. (iii) DBU, *n*-pentanol, Zn(CH_3_COO)_2_, 140 °C. (iv) Toluene, NaH, 120 °C.

Scheme 1 exhibits the synthetic route of peripherally substituted zinc and diaxially substituted silicon phthalocyanines 4 and 5. Firstly, the 1,3-dipolar cycloaddition reaction of azidobenzene and propargyl alcohol gave the starting material compound 1 in 89% yield. Secondly, 6-((1-phenyl-1*H*-1,2,3-triazol-5-yl)methoxy)hexan-1-ol (2) was synthesized by treating (1-phenyl-1*H*-1,2,3-triazol-5-yl)methanol^32^ (1) with 6-chlorohexan-1-ol in ethanol at reflux temperature utilizing NaOH as the base. Thirdly, (1-phenyl-1*H*-1,2,3-triazol-5-yl)methanol (1) was treated with 4-nitrophthalonitrile in the presence of anhydrous K_2_CO_3_, giving phthalonitrile derivative containing 1,2,3-triazole group (3). Lastly, targeted peripherally substituted zinc (4) and axially substituted silicon complex (5) were prepared using starting materials 2 and 3. For zinc complex, the reaction in the presence of DBU or 1,5-diazabicyclo[4.3.0]non-5-ene (DBN) either in *n*-hexanol or in bulk is most efficient in comparison to another method. Therefore, ZnPc complex (4) was obtained by utilizing the anhydrous metal salt [Zn(CH_3_COO)_2_] in *n*-hexanol in the presence of DBU at reflux temperature, the reaction color turned dark blue and the reaction was stopped. To obtain a precipitate, the mixture was washed with different amounts of water and methanol. The new axially disubstituted SiPc complex (5) was prepared by heating from silicon phthalocyanine dichloride (SiCl_2_Pc) and 6-((1-phenyl-1*H*-1,2,3-triazol-5-yl)methoxy)hexan-1-ol in the presence of NaH in toluene at 120 °C for 6 h.

The complexes 4 and 5 are generally soluble in organic solvents and can be readily purified by column chromatography using silica gel or Al_2_O_3_. The structures of the newly synthesized and well-purified compounds were elucidated by UV-Vis, FT-IR, ^1^H-NMR, ^13^C-NMR and MS spectroscopic methods. The results show that the synthesized compounds are coherent with the proposed structures.

The presence of 1,2,3-triazole groups in compound 2, phthalonitrile (3) and its zinc and silicon complexes (4 and 5) were confirmed by FT-IR analysis. Unlike 1,2,4-triazole groups, 1,2,3-triazole groups have stretching vibrations belonging to –N=N groups instead of –C=N, and they appeared around at around 1450 cm^−1^ like compound 2 in the FT-IR spectrum. Furthermore, the peak at 1352 cm^−1^ of the compound (2) containing 1,2,3-triazole group proved the presence of –C–N bonds in the structure (Fig. S1†). For compound 3, stretching vibrations of –C≡N at 2230 cm^−1^, aliphatic –C–H at 2971–2872 cm^−1^ and –C–N at 1309 cm^−1^ appeared at the expected frequencies. Also, the formation of compound 3 was confirmed by the disappearance of the –OH bands at 3372 cm^−1^ for 1 (Fig. S5†). Cyclotetramerization of the dinitrile 3 was confirmed by the absence of the sharp –C≡N vibration around 2230 cm^−1^ after zinc phthalocyanine complex (4) formation (Fig. S9†). Similarly, the formation of silicon phthalocyanine 5 was confirmed by the disappearance of the –OH band at 3372 cm^−1^ for 2 in the FT-IR spectrum of silicon phthalocyanine 5 (Fig. S12†). Generally, FT-IR spectra for both zinc complex 4 and silicon complex 5 support the structures of the complexes.

The ^1^H-NMR spectra are also compatible with the structures of the synthesized compounds. The ^1^H-NMR spectra of compounds 2–5 in CDCI_3_ gave the characteristic signals for the structures as expected. In the ^1^H-NMR spectrum of compound 2, one proton belonging to the 1,2,3-triazole ring was observed at 7.99 ppm as a singlet. Benzene protons were observed as multiplet at 7.69–7.47 ppm integrating five protons. Aliphatic protons were observed at 4.83–1.30 ppm (Fig. S2†). In ^13^C-NMR spectrum of compound 2, the aromatic and aliphatic carbons appeared at 137.16, 129.99 (overlapped 2C signals), 129.06, 120.77 (overlapped 2C signals), 120.39, 70.86, 64.46, 62.98, 32.84, 29.79, 26.13, 25.76 ppm (Fig. S3†). In the ^1^H-NMR spectrum of compound 3, one proton belonging to the 1,2,3-triazole ring was observed at 8.11 ppm as a singlet. Benzene protons were observed as multiplet at 7.75–7.48 ppm integrating eight protons. One singlet peak for OCH_2_ integrating two protons observed at 5.40 ppm (Fig. S6†). In ^13^C-NMR spectrum of compound 3, fifteen aliphatic and aromatic carbons and two nitrile carbons appeared at 161.38, 142.80, 136.88, 135.61, 130.14 (overlapped 2C signals), 129.51, 121.88, 120.87 (overlapped 2C signals), 120.55, 119.74, 117.72, 115.78, 115.37, 108.16, 62.74 ppm (Fig. S7†). The ^1^H-NMR spectrum of 4 is somewhat broader than the corresponding chemical shifts in the dinitrile derivative 3. ^1^H-NMR spectra of phthalocyanines are of broad nature, because of the presence of four positional isomers which tend to show similar chemical shifts. The second reason for broad signals is that phthalocyanines show an equilibrium of aggregation and disaggregation. In the ^1^H-NMR spectrum of zinc complex 4, four protons belonging to the 1,2,3-triazole ring was observed at 9.18 ppm as a singlet. Aromatic benzene protons of phthalocyanine and 1,2,3-triazole rings were observed as multiplet at 8.84–7.46 ppm. One singlet peak for OCH_2_ integrating eight protons observed at 5.75 ppm (Fig. S10†). The ^1^H-NMR measurement of silicon phthalocyanine complex 5 showed the expected total number of aliphatic and aromatic protons, confirming the purity of the complex. The ^1^H-NMR spectrum of silicon phthalocyanine complex 5 showed H_α_ and H_β_ aromatic protons between 9.56 and 8.26 ppm. The other protons belonging to the 1,2,3-triazole ring and benzene protons were observed between 7.92 and 7.26 ppm. Aliphatic protons were observed 4.61–(−1.76) ppm (Fig. S13†). ^1^H-NMR spectrum of 5, Si–O–CH_2_ has shifted negative area (−1.76 ppm) because of magnetic anisotropy of phthalocyanine ring.^33^

**Fig. 1:**
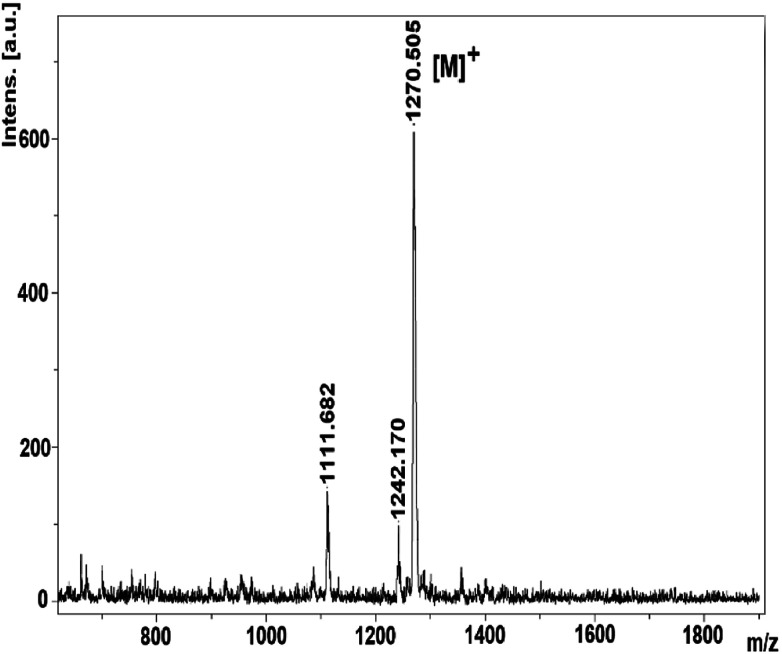
The mass spectrum of the 1,2,3-triazole group substituted zinc phthalocyanine (4).

The mass spectra of derivatives, which were obtained by MALDI-TOF techniques, confirmed the proposed structures. By using the instrument's reflectron mode to compare the theoretical and experimental monoisotopic *m*/*z* values of the compounds and starting materials, the highly resolved signals of each species in the MALDI-TOF mass spectra were well obtained. After evaluation of the MALDI-TOF mass spectra, it was found that the desired starting compounds and complexes were successfully purified using the experimental methods expressed in this study. In addition, it has been found that the synthesized compounds are sufficiently stable to detect structures without significant fragmentation under the conditions of MALDI-MS. The molecular ion peaks were observed at *m*/*z*: 275.85 [M]^+^ for 2 (Fig. S4†), 301.43 [M]^+^ for 3 (Fig. S8†), *m*/*z*: 1270.50 [M]^+^ for 4 (Fig. 1), 1095.98 [M + 6H]^+^ for 5 (Fig. S14†).

### 3.2. Photophysical and photochemical studies

#### 3.2.1. Ground state electronic absorption spectra and aggregation studies

**Fig. 2:**
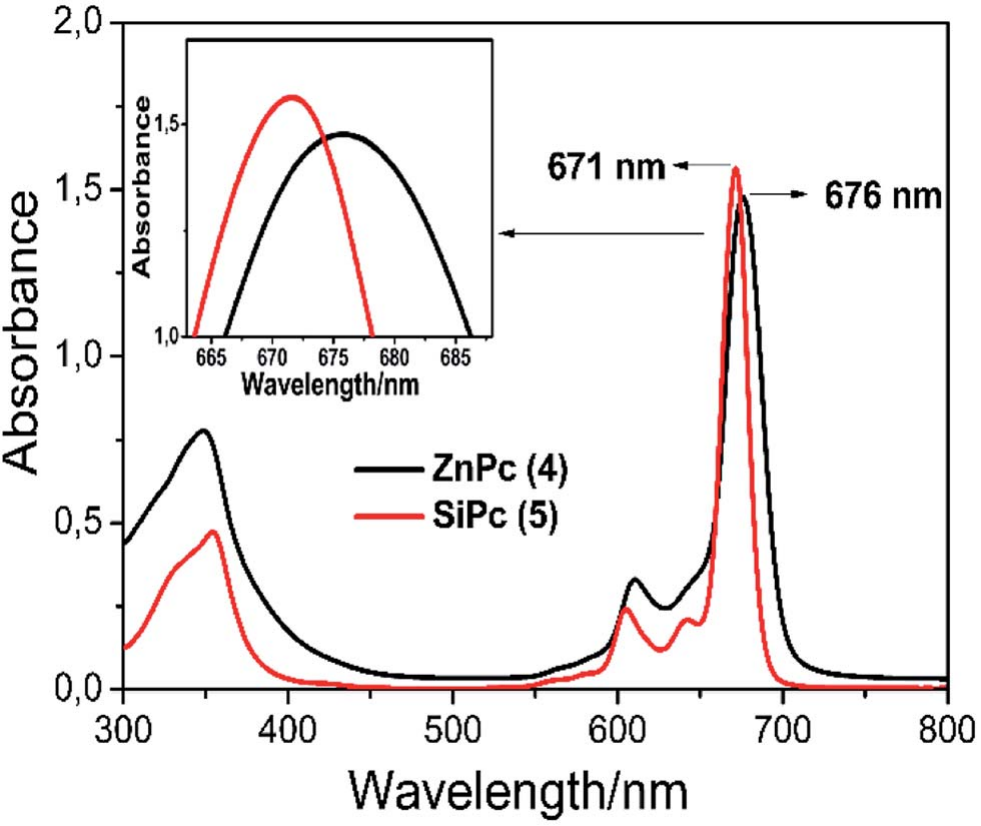
Absorption spectra of complexes 4 and 5 in THF (∼2 × 10^−5^ mol dm^−3^).

UV-Vis spectra of the phthalocyanines shows characteristic absorptions in the Q-band region at around 680–700 nm, attributed to the π–π* transition from the HOMO to the LUMO of the Pc ring, and in the B band region (UV region) at around 350–360 nm, arising from the deeper π–π*transitions. As it is expected that the UV-Vis absorption spectra of the blue colored axial silicon and green colored peripheral zinc phthalocyanine complexes in THF exhibited two main peaks, the characteristic ligand centered π–π* transitions of a monomeric phthalocyanines 4 and 5 with the B-band and Q-band maxima at 347, 353 nm (log *ε* = 4.77 and 4.56) and 676, 671 nm (log *ε* = 4.86 and 4.89), respectively (Fig. 2).

It is known that the central metal ions in the phthalocyanine macrocycle can change the maximum value of the Q band maximum. In porphyrins present in the same family as phthalocyanine complexes, the blue shift of the Q band differs according to the ionic radius of the central metal ions and the electronegativity. Briefly, a larger electronegativity value or smaller ionic radius results in a shift to blue wavelength.^34^ It can be seen that the *λ*_max_ of complex 5 exhibits a blue shift with respect to complex 4 owing to the Si(iv) ion has a smaller radius and larger electronegativity than the Zn(ii) ion. As a result, the wavelengths of the Q-band absorption of the MPcs follow the order of Zn > Si (Fig. 2).

**Fig. 3:**
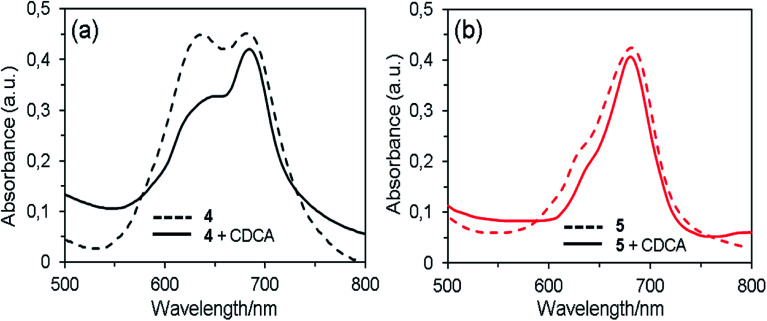
UV-Vis absorption spectra of 4 (a) and 5 (b) without and with 1 mM CDCA on TiO_2_ film.

When the dyes are adsorbed on the TiO_2_ film without chenodeoxycholic acid (CDCA) as the co-adsorbent, the Q-band absorption maxima are red shifted by ∼10 nm compared with those in THF solution (Fig. 3). This result indicates that the interaction of 1,2,3-triazole moiety and TiO_2_ surface, which is similar to that reported in the interaction of the pyridine ring of the D–π–A fluorescent dyes with TiO_2_ surface.^6^ The spectrum of dye 4 exhibits the broadened Q-band and a new peak at around 635 nm, demonstrating the existence of molecular aggregates on the semiconductor surface.^35^ By the addition of the co-adsorbent to the dye solution, the absorption intensity of the new band relative to that of the Q-band remarkable decreased, which suggests that the aggregation of dye 4 on the TiO_2_ surface was suppressed by CDCA co-sensitization. In contrast, the spectra of dye 5 with and without CDCA show that there is little or no dye aggregation, indicating that the aggregate formation can be effectively hindered by the steric effect of long and flexible axial 1,2,3-triazole group in the molecule.

**Fig. 4:**
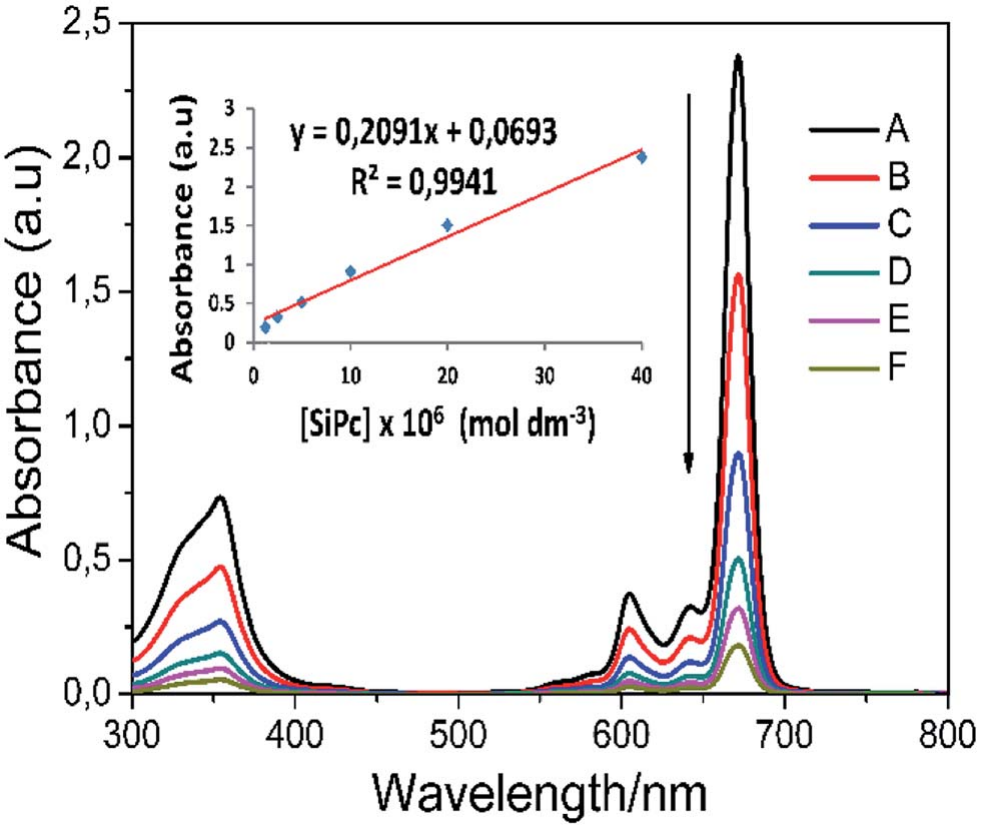
Electronic absorption spectra of complex 5 in THF at different concentrations: (A) 40 × 10^−6^, (B) 20 × 10^−6^, (C) 10 × 10^−6^, (D) 5 × 10^−6^, (E) 2.5 × 10^−6^ (F) 1.25 × 10^−6^ mol dm^−3^. The inset shows the calibration plot for Q band maximum.

Aggregation can often be expressed as the superposition of monomers, dimers, and rings in the solvent medium. There are lots of parameters for aggregation in phthalocyanines; concentration, the nature of the solvent, substituents present at the periphery or non-periphery, the metal ion at the macrocyclic core, and the operating temperature. In order to better examine the solubility and aggregation properties of the complexes, the aggregation study was also performed at ambient temperature in THF. The absorption spectra of phthalocyanines 4 and 5 were also obtained at different concentrations and Fig. 4 shows the results for complex 5. Axial substituted silicon phthalocyanine and peripheral substituted zinc phthalocyanine did not aggregate in THF at studied concentrations (Fig. S15†). As the concentration increased, a new band did not form on the high energy side owing to the formation of aggregate species, and the absorption intensity of the Q band was consistent with the Lambert–Beer's law (inset of Fig. 4).

**Fig. 5:**
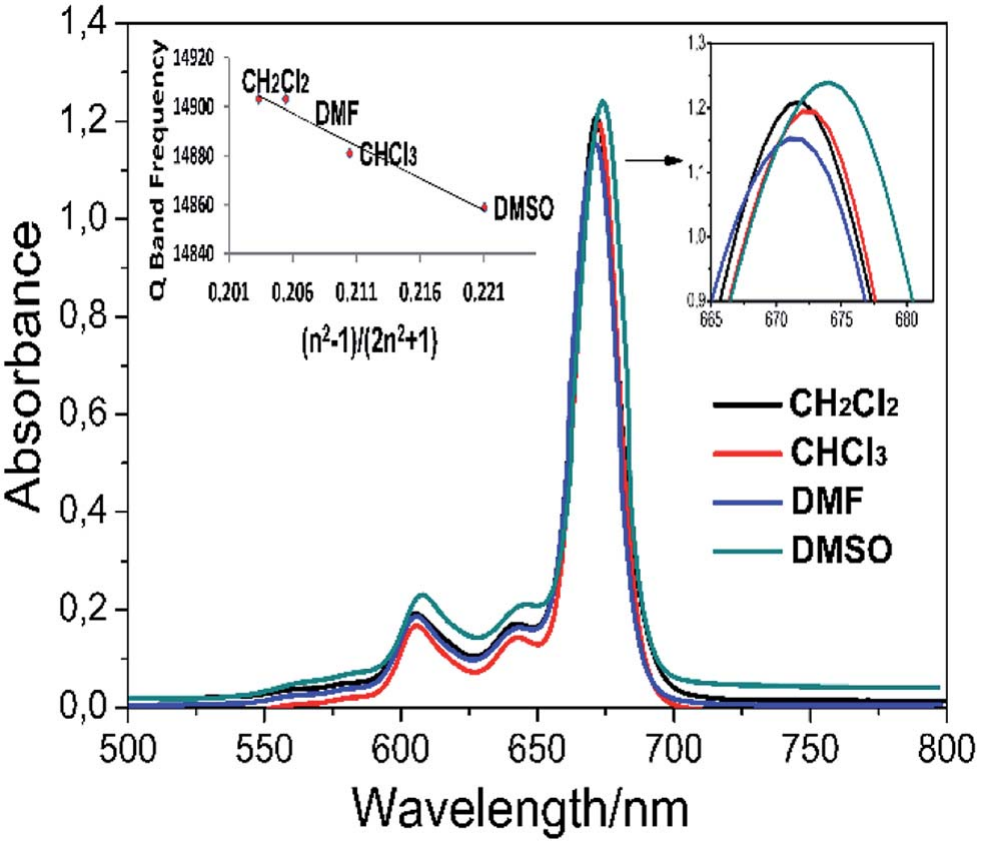
Absorption spectra of complex 5 in various solvents (∼15 × 10^−6^ mol dm^−3^). The inset exhibits a plot of the Q band frequency of complex 5 against the function (*n*^2^ − 1)/(2*n*^2^ + 1), where *n* is the solvents' refractive index.

Solvents may interact with absorbing species in the solution, which changes the absorption wavelength. This interaction depends on the polarity of the solvent, the refractive index, the coordination power and the chemical structure of the solute. In general, the Q band wavelength of the phthalocyanine in the solvent shows red-shifting by increasing the refractive index of the solvent. This can best be defined by the Franck–Condon principle. According to this principle, the refraction index and the polarity of the solvent suggest that the species in the solvent can change the absorption wavelength. Thus, it is estimated that the Q band wavelength in a specific solvent is only related to the refractive index of the solvent, unless the solvent reacts with the species in the solution or induces any reaction. The electronic absorption spectra of complex 5 in different organic solvents (DMSO, DMF, CH_2_CI_2_, and CHCI_3_) and the plot of the Q band frequency *versus* the function (*n*^2^ − 1)/(2*n*^2^ + 1), where *n* is the refractive index of the solvent,^36^ are shown in Fig. 5. It can be seen that the Q band frequencies exhibit a linearly dependent on this function which proposes the Q band wavelength changes directly as the solvation rather than coordination.

#### 3.2.2. Fluorescence studies

**Fig. 6:**
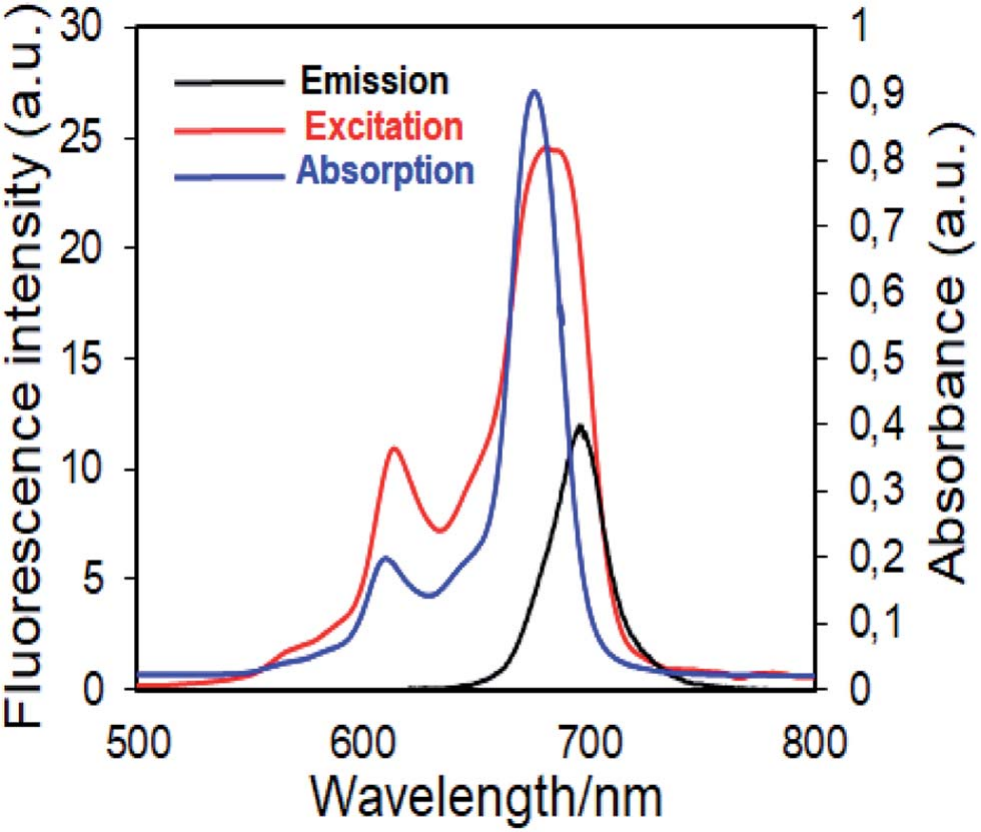
Electronic absorption, fluorescence emission and excitation spectra of 4 in DMSO. (Excitation wavelength = 697 nm.)

**Fig. 7:**
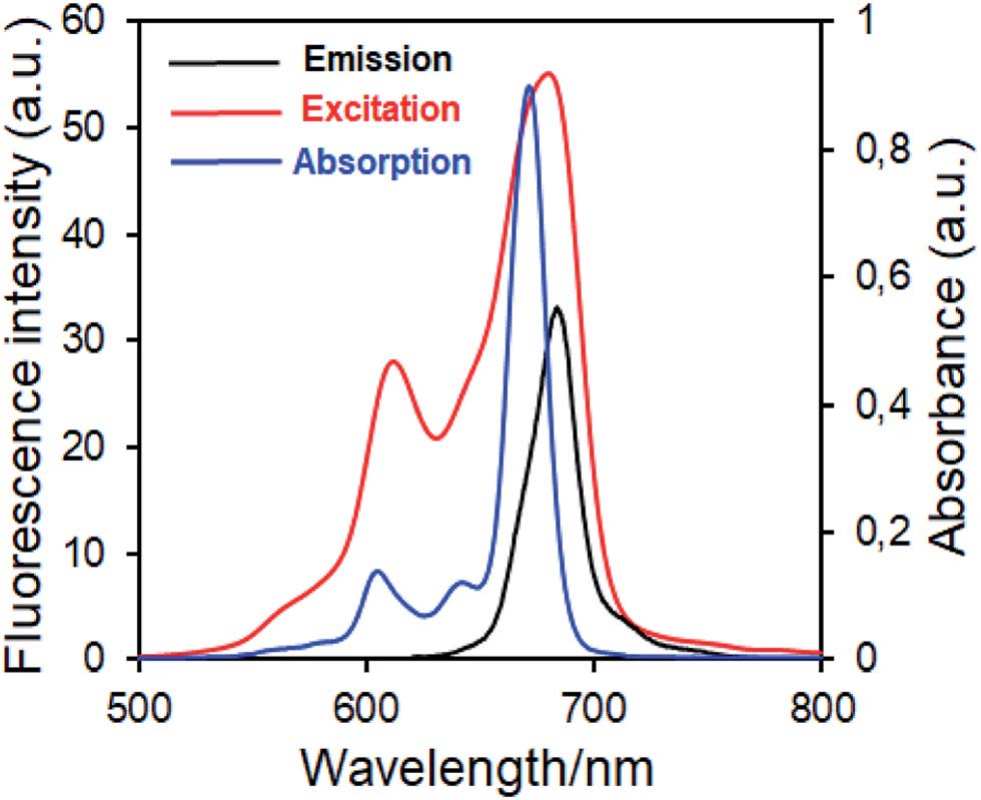
Electronic absorption, fluorescence emission and excitation spectra of 5 in DMSO. (Excitation wavelength = 686 nm.)

First studies to determine a photosensitizer for use in photodynamic therapy are the determination of fluorescence behavior and fluorescent quantum yields. Fluorescence quantum efficiency (*Φ*_F_) determines the efficiency of the fluorescence process. In this context, the fluorescence emission, excitation and absorption spectra of novel synthesized zinc(ii) phthalocyanine complex (4) and axially disubstituted silicon(iv) phthalocyanine complex (5) examined and these complexes showed similar fluorescence behavior in DMSO (Fig. 6 for complex 4, Fig. 7 for complex 5). The UV-Vis absorption, emission, excitation and fluorescence quantum yield (*Φ*_F_) of the complexes 4 and 5 are given in Table 1.

**Table 1:** Optical, photophysical and photochemical properties of the phthalocyanines complexes in DMSO

Dye	*λ*_max_ a (nm)	(log *ε*)	Excitation *λ*_Ex_ (nm)	Emission *λ*_Em_ (nm)	Stokes shift *Δ*_Stokes_ (nm)	Fluorescence quantum yield (*Φ*_F_)	Singlet oxygen quantum yield (*Φ*_Δ_)
4	676	4.86	681	691	15	0.14	0.76
5	671	4.89	679	679	8	0.28	0.32

a Absorption maximum wavelength (*λ*_max_) in THF solution.

Fluorescence emission maxima were observed at 691 nm for complex 4, 679 nm for compound 5 in DMSO. Stokes shifts from the complexes were detected to be in the same region as the standard phthalocyanines. However, in the case of wavelength results, the excitation spectra of the complexes 4 and 5 were slightly red-shifted when compared to the absorption spectra, suggesting that the nuclear configurations change the following excitation.

Fluorescence quantum yields of synthesized phthalocyanine complexes were determined using a comparative method. The standards used were ZnPc (*Φ*_F_ = 0.18 (ref. 37)) for complex 4 in DMSO. The 1,2,3-triazole group containing silicon(iv) complex (5) showed higher *Φ*_F_ values than zinc(ii) complex (4). Although the *Φ*_F_ value of 5 (SiPc) was higher than standard unsubstituted ZnPc and is lower than unsubstituted SiPc(Cl)_2_ in DMSO.^38^ Furthermore, the silicon complex (5) shows a higher fluorescence quantum yield than zinc complexes containing 1,2,4-triazole group in the literature,^29^ showing the superiority of the silicon complex over complexes containing similar groups. Fluorescence results revealed that phthalocyanines having the similar substituent; the silicon complex (5) showed a higher fluorescence quantum yield than the zinc complex (4) due to relatively less aggregation in DMSO solution.^38^

#### 3.2.3. Singlet oxygen generation capability studies

It is known that diphenyl isobenzofuran (DPBF) is a single oxygen quencher which causes the formation of endoperoxide species with singlet oxygen in the solution medium and by the chemical addition reaction. The examination of the singlet oxygen capacity of the 1,2,3-triazole group-containing phthalocyanine complexes was carried out in a similar method defined previously in DMSO.^13^

**Fig. 8:**
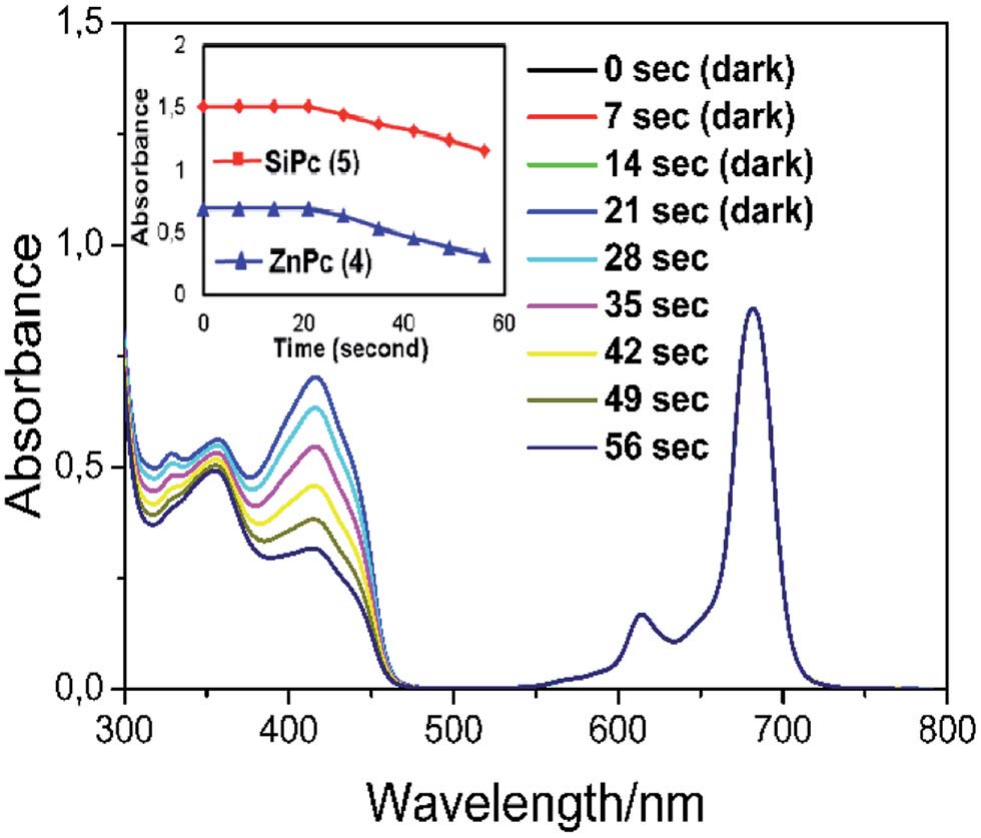
The reaction of singlet oxygen formed by zinc phthalocyanine complex (4) dissolved in DMSO with 1,3-diphenyl-isobenzofuran (DPBF). For the first 21 s, the solution was kept in the dark; thereafter, it was irradiated with a light source (650 nm cut-on filter) for 35 s. The total volume of the solution was set to be 3 mL. The absorption spectra were recorded every 7 seconds. The inset shows plot for DPBF absorbance in phthalocyanine solutions (4 and 5) *versus* irradiation time (second).

In order to show that the peripherally and axially 1,2,3-triazole group substituted zinc and silicon phthalocyanines are acceptable as useful photosensitizers, the phthalocyanines solution were prepared in a DPBF containing a DMSO solution. Careful control experiments have been carried out to remove other potential sources of media that can effect the absorption decrease. The spectral changes during singlet oxygen determination for complex 4 were given in Fig. 8. No significant change was observed in the absorption spectrum of phthalocyanines when kept in the dark for 21 seconds. (Fig. 8 for zinc complex 4, Fig. S16† for silicon complex 5.)

Furthermore, the absorption peak belonging to the capture compound DPBF disappeared rapidly within 56 seconds in the irradiation red-light (with 650 nm cut-on filter) on phthalocyanine solutions. The Q band intensity of studied zinc and silicon phthalocyanine complexes did not show any changes during the *Φ*_Δ_ determinations, confirming that this complex did not degrade using light irradiation during singlet oxygen studies. The *Φ*_Δ_ values were found 0.76 for zinc complex (4) and for 0.32 silicon complex (5). The *Φ*_Δ_ value of zinc phthalocyanine complex is 0.76 and this value is higher than the *Φ*_Δ_ value (*Φ*_Δ_ = 0.67 (ref. 39)) of respective unsubstituted ZnPc reference in DMSO. Similarly, the *Φ*_Δ_ value of silicon phthalocyanine complex is 0.32 and this value is higher than the *Φ*_Δ_ value (*Φ*_Δ_ = 0.15 (ref. 38)) of respective unsubstituted SiPc(Cl)_2_ reference in DMSO. Compared to complexes containing similar triazole substituents in the literature,^29,40^ the zinc complex (4) exhibited a slightly higher singlet oxygen quantum yield value. Consequently, the substitution of the Pc core with 1,2,3-triazole groups increased the generation of singlet oxygen which is very functional for the usability of these Pcs as photosensitizers for PDT applications.

### 3.3. Electrochemical studies

**Fig. 9:**
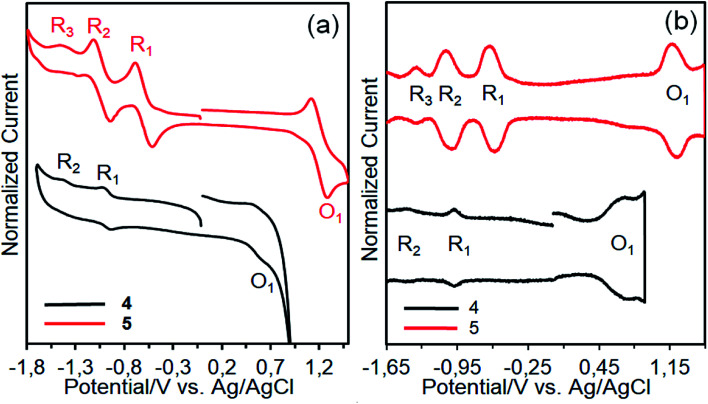
CVs (a) and SWVs (b) of dyes 4 and 5 on a GCE in TBABF_4_/DMSO.

The redox behaviors and energy levels of dyes 4 and 5 were investigated by using cyclic voltammetry (CV) and square wave voltammetry (SWV) measurements (Fig. 9) and the relevant data are listed in Table 2, along with some optical properties. It can be seen that dyes 4 and 5 show three and four redox processes, respectively. Compared with dye 4, dye 5 is not only more hardly oxidized but also more easily reduced owing to the tetra positive nature of silicon.^41^ The redox processes of dye 4 are considerably broad, implying that the presence of aggregated species due to the differences in the redox potentials of aggregated and nonaggregated species.^42^ In contrast, the redox processes of dye 5 are not broad. This may be due to the absence of aggregated species owing to its nonplanar nature. On the other hand, the oxidation potentials (O_1_) corresponding the HOMO levels of the dyes are more positive than that of the I^−^/I_3_^−^ redox couple (0.4 V *vs.* NHE), guaranteeing efficient dye regeneration.^43^ The LUMO value of dye 4 is much more negative than the conduction band (CB) of TiO_2_ (−0.5 V *vs.* NHE), insuring efficient electron injection from the excited state of the dye to the TiO_2_ surface. However, the LUMO level of dye 5 is slightly more negative than the CB of TiO_2_ and electron injection might be less effective.^44^

**Table 2:** Electro-optical properties of the dyes

Dye	*λ*_onset_ a (nm)	*E*_0–0_ b (eV)	*E*_HOMO_ c (V)	*E*_LUMO_ d (V)
4	699	1.77	0.65	–1.12
5	687	1.80	1.26	–0.54

a Absorption onset wavelength (*λ*_onset_) in THF solution.

b The *E*_0–0_ (band gap) was calculated from the *λ*_onset_ using *E*_0–0_ = 1240/*λ*_onset_.

c The *E*_HOMO_ was taken from the *E*_1/2_ of the O_1_ process in Fig. 9. The *E*_1/2_ of Fc/Fc^+^ was found to be 0.54 V, *vs.* Ag/AgCl. By comparing this value with that of 0.63 V *vs.* NHE, the potentials *vs.* Ag/AgCl can be converted to that *vs.* NHE by adding a value of 0.09 V.

d The *E*_LUMO_ was estimated with the formula *E*_LUMO_ = *E*_HOMO_ − *E*_0–0_.

### 3.4. Photovoltaic studies

**Fig. 10:**
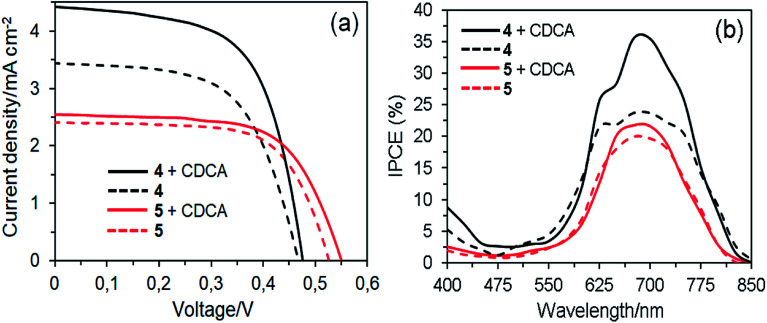
*J*–*V* curves (a) and IPCE spectra (b) of the DSSCs based on dyes 4 and 5 with and without CDCA.

Fig. 10 shows the *J*–*V* curves and IPCE spectra of the DSSCs based on 4 and 5 dyes fabricated in the absence and presence of 1 mM CDCA and their photovoltaic parameters are listed in Table 3. All the parameters presented are at optimum conditions. In the absence of CDCA, the short circuit current density (*J*_SC_) of 3.44 mA cm^−2^ and power conversion efficiency (PCE) of 0.96% for dye 4 based DSSC are slightly higher than dye 5 (*J*_SC_ = 2.41 mA cm^−2^ and PCE = 0.84%). The photovoltaic performance of the former could be attributed to its red-shift absorption and higher dye loading on the TiO_2_ surface.^45^ This result revealed that the presence of four anchoring groups in the molecular structure of dye 4 results in a more efficient electron injection process into the TiO_2_ than its counterpart of two anchoring groups because the former provides a more effective binding onto the TiO_2_ surface, which is consistent with the increased dye loading.^46^ On the other hand, the dye 5 molecule can be bonded to the TiO_2_ surface by only one of the axial anchoring groups. The open-circuit voltage (*V*_OC_) of the dye 5 based device is higher than the dye 4 cell, indicating the suppression of charge recombination. Apparently, one of the axial groups, which is unable to bind to the TiO_2_ surface, favors to the improvement of *V*_OC_ because this bulky group can block the I_3_^−^ ions in the electrolyte from approaching the TiO_2_ surface, thus reducing the charge recombination rate.^47^ In the presence of CDCA, the cell based on dye 4 showed the highest PCE of 1.30%, which is a 35% improvement compared with the cell without CDCA. It indicates that this dye forms strong aggregates on the TiO_2_ surface in the absence of CDCA. In contrast, co-adsorption of dye 5 with CDCA increased the PCE (0.90%) of DSSC by around 7%, suggesting that the degree of molecular aggregations was very few. This result demonstrates that the introduction of sterically hindered substituents such as long and flexible axial 1,2,3-triazole units in the dye structures can expeditiously suppress aggregation, owing to disturbance of the π–π stacking.^48^

**Table 3:** Photovoltaic parameters of the DSSCs using ethanol/THF (1/1) as a dye-loading solvent mixture

Dye	CDCA (mM)	IPCE integrated current density (mA cm^−2^)	*J*_SC_ (mA cm^−2^)	*V*_OC_ (V)	FF	PCE (%)	Dye-loaded amount (mol cm^−2^)
4	0.00	3.48	3.44	0.467	0.60	0.96	3.69 × 10^−8^
4	1.00	4.39	4.42	0.475	0.62	1.30	3.07 × 10^−8^
5	0.00	2.40	2.41	0.526	0.66	0.84	2.86 × 10^−8^
5	1.00	2.48	2.53	0.550	0.65	0.90	2.68 × 10^−8^

The difference in *J*_SC_ was further confirmed by IPCE spectra, as a function of the incident light wavelength (Fig. 10b and Table 3). In good agreement with absorption spectra on the TiO_2_ films, the IPCE spectra for DSSCs based on dye 4 are broader than those of dye 5. In the absence of CDCA, the highest IPCE values of DSSCs based on dyes 4 and 5 are 23% and 19% at about 690 nm, respectively. However, the highest IPCE values of devices based on dyes 4 and 5 with the co-adsorbent represent 52% and 16% of improvement compared with those of the devices fabricated without the co-adsorbent. This means that the latter is less affected by aggregation issues. Nevertheless, compared with dye 4, dye 5 is unfavorable for photovoltaic applications due to the axial groups in the molecules significantly reduce the amount of dye loading onto the TiO_2_ surface, resulting in lowering of the photovoltaic performance.^49^

**Fig. 11:**
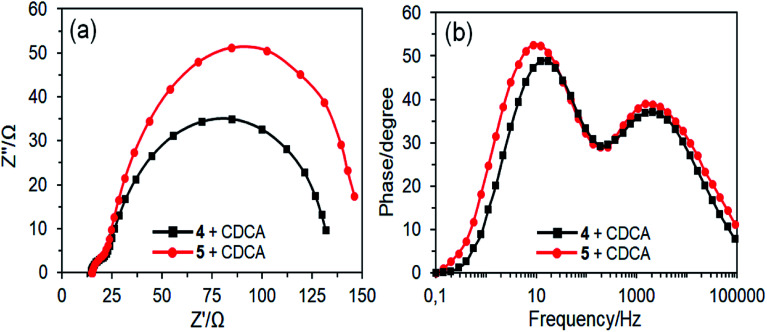
Nyquist (a) and Bode (b) plots of DSSCs based on dyes 4 and 5 with CDCA in the dark.

The *J*–*V* curves also display that the *V*_OC_ values for DSSCs fabricated with CDCA are slightly improved as compared to those of without CDCA. The improvement of *V*_OC_ can be explained by the co-adsorbent occupation of the free TiO_2_ surface and thereby to suppress the charge recombination.^50^ Electrochemical impedance spectroscopy (EIS) analysis was performed to further verify the *V*_OC_ values of the DSSCs in the presence of CDCA (Fig. 11). The Nyquist plots have two semicircles and the right semicircle exhibits the charge recombination resistance between the TiO_2_ and the electrolyte (Fig. 11a). The radius of the right semicircle of DSSC based on dye 5 is higher than that of dye 4, which is agreement with their *V*_OC_ values. The frequency maxima (*f*) of the left peaks in the Bode plots for DSSCs based on dyes 4 and 5 are 12.19 and 8.65 Hz, respectively (Fig. 11b). The corresponding electron lifetimes (*τ*_e_), which can be estimated using equation *τ*_e_ = 1/2π*f*,^51^ are 13.06 and 18.4 ms, respectively, which is consistent with the *V*_OC_ values.

## 4. Conclusion

As a consequence, in this work, the preparation, spectral characterization, aggregation, photophysical, singlet oxygen capability, electrochemical and photovoltaic properties of novel peripherally substituted zinc and axially disubstituted silicon phthalocyanine complexes are presented for the first time. The photophysical and singlet oxygen generation properties of axially substituted silicon and peripherally substituted zinc phthalocyanine complexes were investigated in DMSO. The 1,2,3-triazole group containing silicon(iv) complex (5) showed higher *Φ*_F_ values than zinc(ii) complex (4) and standard unsubstituted ZnPc. Also, the studied zinc complex (4) is produced efficiently singlet oxygen (*Φ*_Δ_ = 0.76) in DMSO. These photophysical and photochemical results indicate that the synthesized phthalocyanine complexes (especially zinc complex) may be alternative to clinically approved photosensitizers with good solubility, red-shifts in the absorption spectrum, non-aggregating behaviors, and acceptable singlet oxygen quantum yields. Compared to complex 5, complex 4 exhibited red-shifted absorption and higher dye loading, which is beneficial for absorbing more photons and thus generating high photocurrent. On the other hand, the power conversion efficiencies of solar cells based on complexes 4 and 5 with the co-adsorbent improved by 35% and 7%, respectively, indicating that the introduction of axial 1,2,3-triazole groups may be able to play an anti-aggregation effect. This study has shown that these zinc and silicon phthalocyanines are promising photosensitizers for both photodynamic cancer therapy and dye-sensitized solar cell applications.

## Conflicts of interest

There are no conflicts to declare.

## Supplementary Material

RA-009-C8RA10665G-s001
