# Supplementary material for: Dual-purpose zinc and silicon complexes of 1,2,3-triazole group substituted phthalocyanine photosensitizers: synthesis and evaluation of photophysical, singlet oxygen generation, electrochemical and photovoltaic properties
Source: RSC Adv. 2019 Apr 8;9(19):10854–64. doi: 10.1039/c8ra10665g (PMC9062642; doi:10.1039/c8ra10665g)
Supplement: RA-009-C8RA10665G-s001 [file RA-009-C8RA10665G-s001.pdf]

Electronic Supplementary Material (ESI) for RSC Advances.
This journal is © The Royal Society of Chemistry 2019

SUPPLEMENTARY INFORMATION

Dual-purpose zinc and silicon complexes of 1,2,3-triazole group substituted phthalocyanine photosensitizers: synthesis and evaluation of photophysical, singlet oxygen generation, electrochemical and photovoltaic properties

Emre Güzel*

Department of Chemistry, Sakarya University, TR54050 Serdivan, Sakarya, Turkey

* Corresponding author. Fax: +90 264 295 74 26. E-mail address: emreguzel_@outlook.com, eguzel@sakarya.edu.tr

1. Materials

All reagents and solvents were of reagent grade quality obtained from commercial suppliers. The homogeneity of the products was tested in each step by TLC. The solvents were stored over molecular sieves. All solvents were dried and purified as described by Perrin and Armarego.[1] (1-phenyl-1H-1,2,3-triazol-5-yl)methanol (**1**) was prepared according to reported procedure.[2] The compound, 2,5-dihydroxybenzoic acid (DHB) was chosen as the best MALDI matrix.

2. Equipment

IR spectra were recorded on a Perkin Elmer Spectrum One FT-IR (ATR sampling accessory) spectrophotometer, electronic spectra on a Scinco S-3100 spectrophotometer. ^{1}H-NMR and ^{13}C NMR spectra were recorded on Agilent VNMRS 300 MHz and the spectrum was referenced internally by using the residual solvent resonances and chemical shifts were reported relative to Me_4Si as internal standard. Mass spectra were measured on a Micromass Quatro LC/ULTIMA LC-MS/MS spectrometer and MALDI-MS of complexes were obtained in dihydroxybenzoic acid as MALDI matrix using nitrogen laser accumulating 50 laser shots using Bruker Microflex LT MALDI-TOF mass spectrometer Bremen, Germany. Elemental analyses were performed in TÜBİTAK Marmara Research Centre.

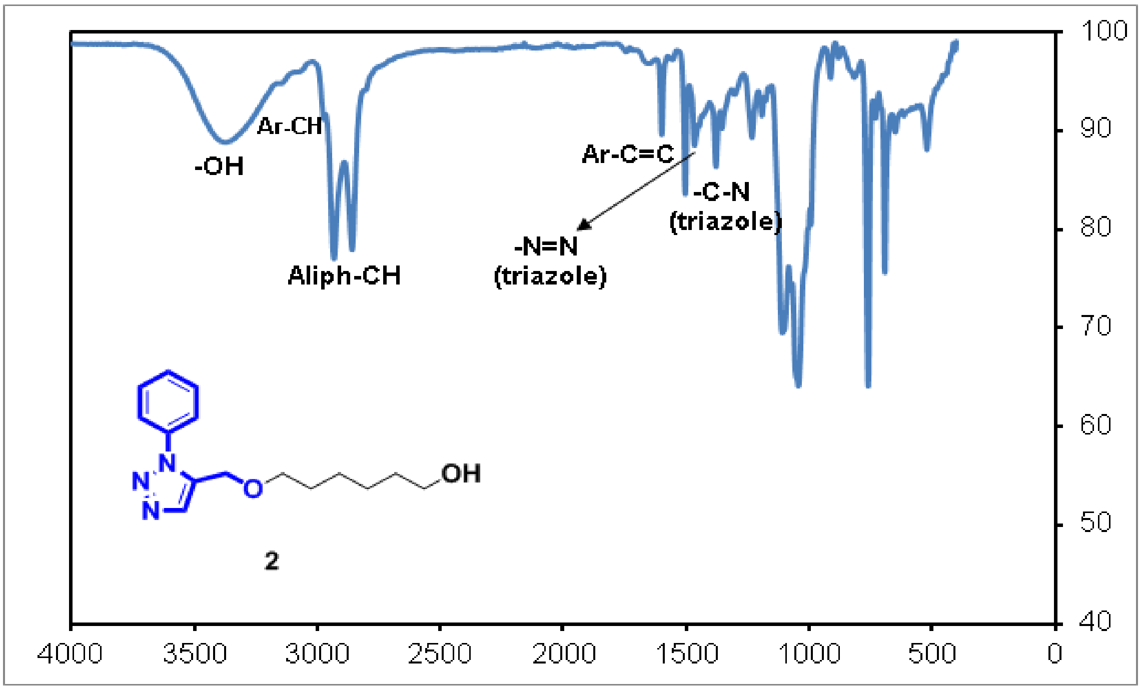

Figure S1. FT-IR spectrum of compound **2**.

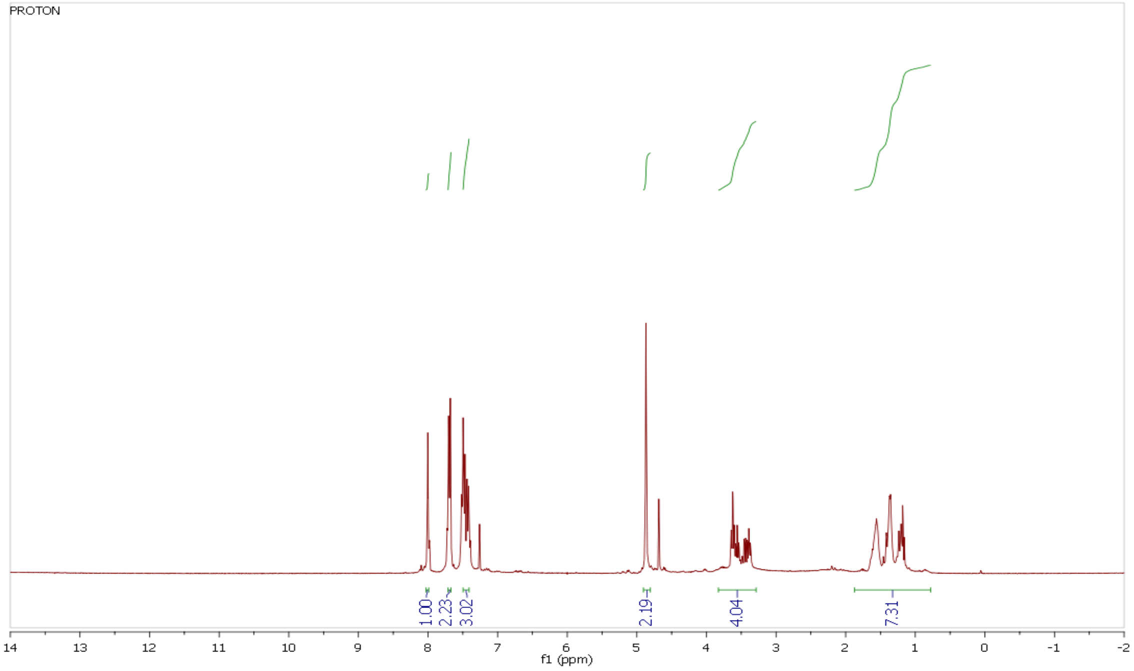

Figure S2. ^{1}H-NMR spectrum of compound **2** in $CDCl_3$.

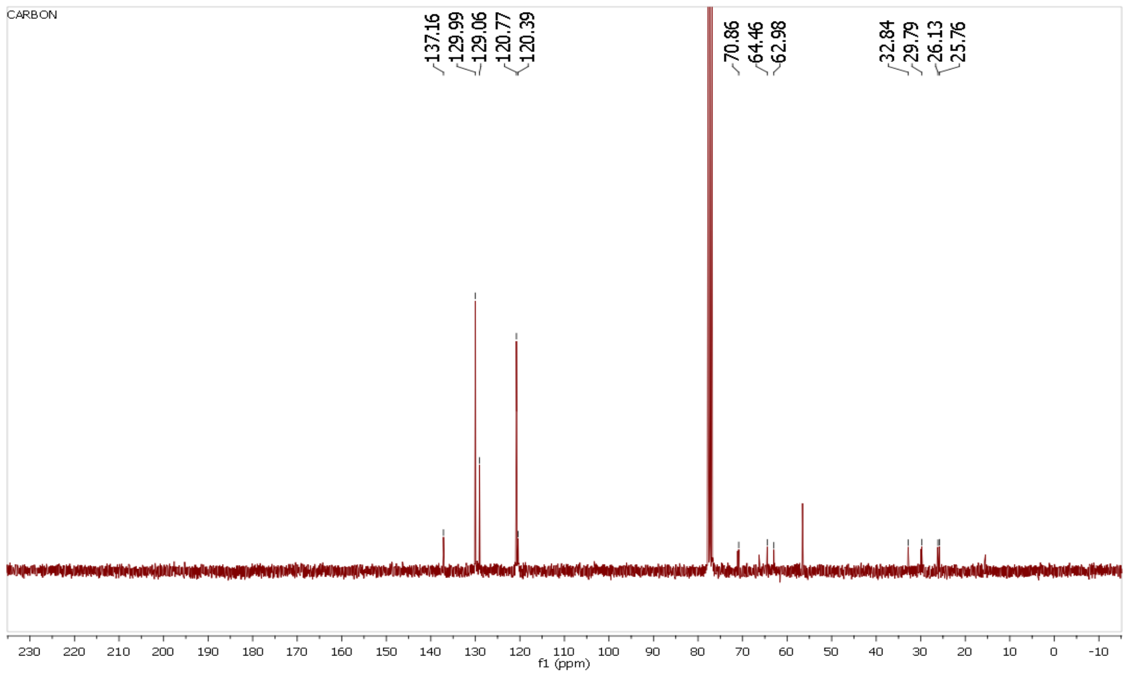

Figure S3. ^{13}C-NMR spectrum of compound **2** in $CDCl_3$.

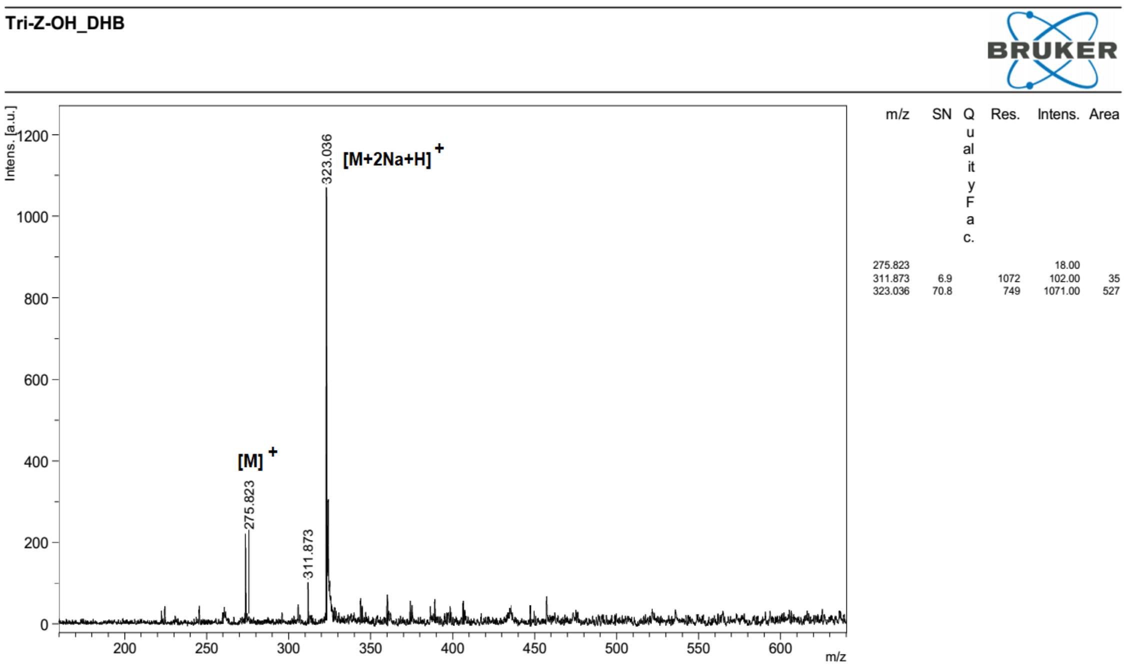

Figure S4. The MALDI-TOF mass spectrum of the compound **2**.

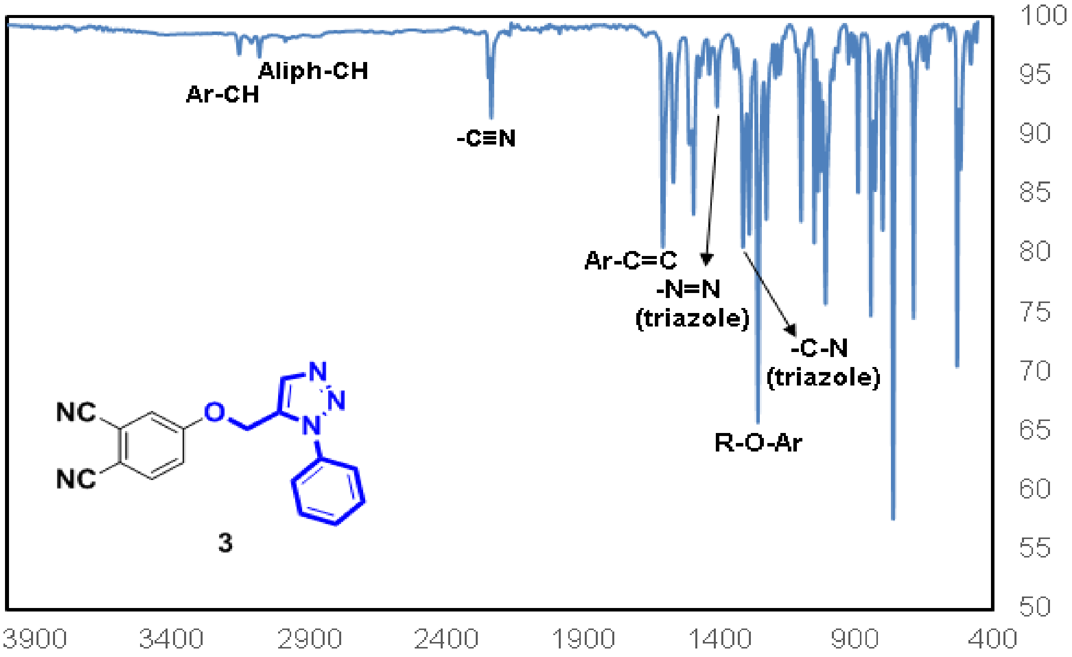

Figure S5. FT-IR spectrum of compound **3**.

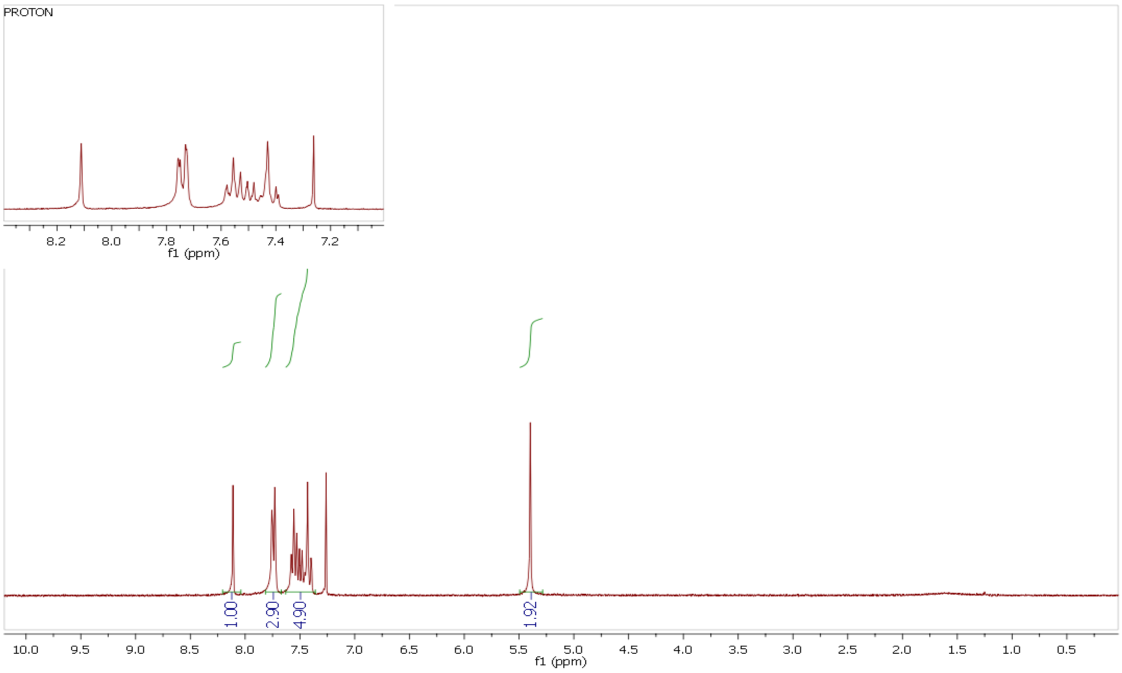

Figure S6. ^{1}H-NMR spectrum of compound **3** in $CDCl_3$.

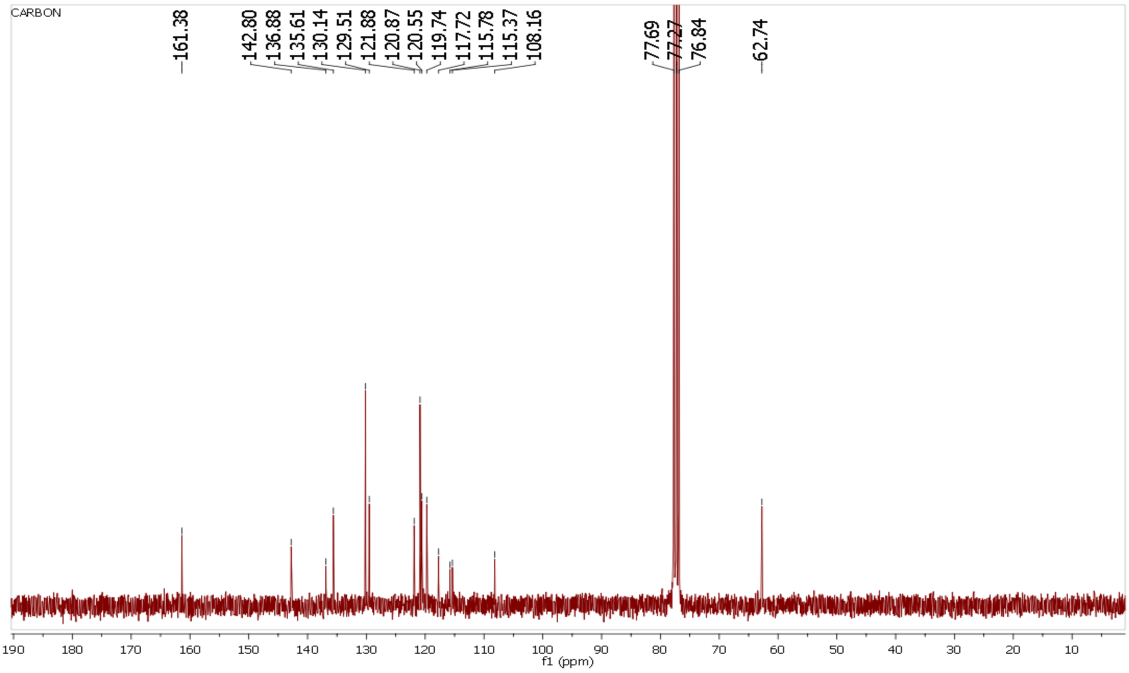

Figure S7. ^{13}C-NMR spectrum of compound **3** in $CDCl_3$.

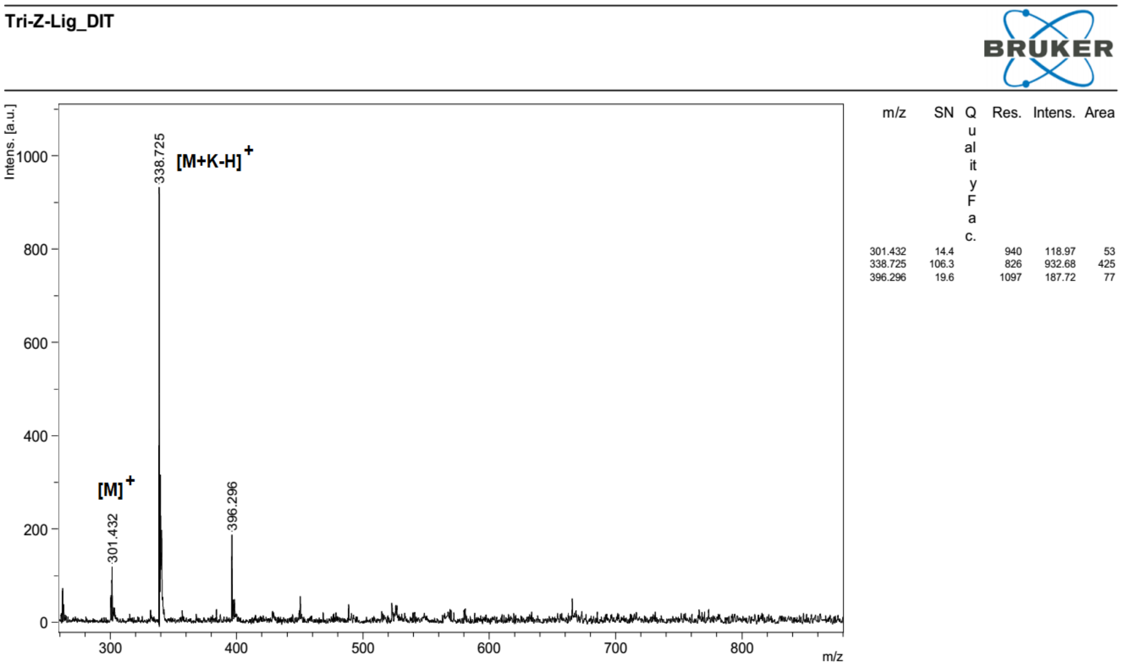

Figure S8. The MALDI-TOF mass spectrum of the compound **3**.

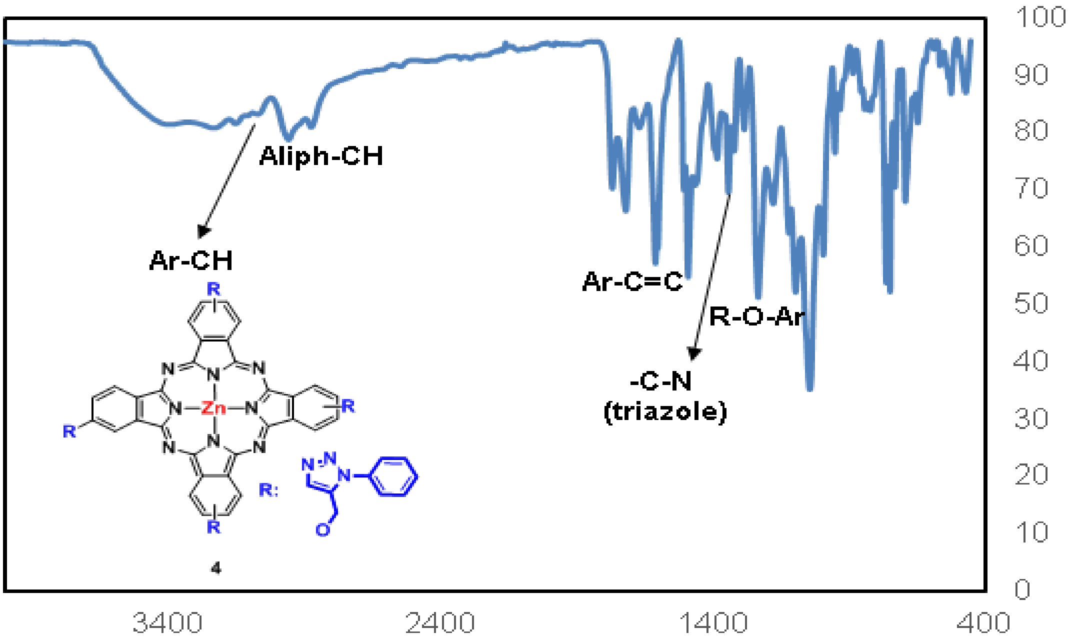

Figure S9. FT-IR spectrum of zinc complex **4**.

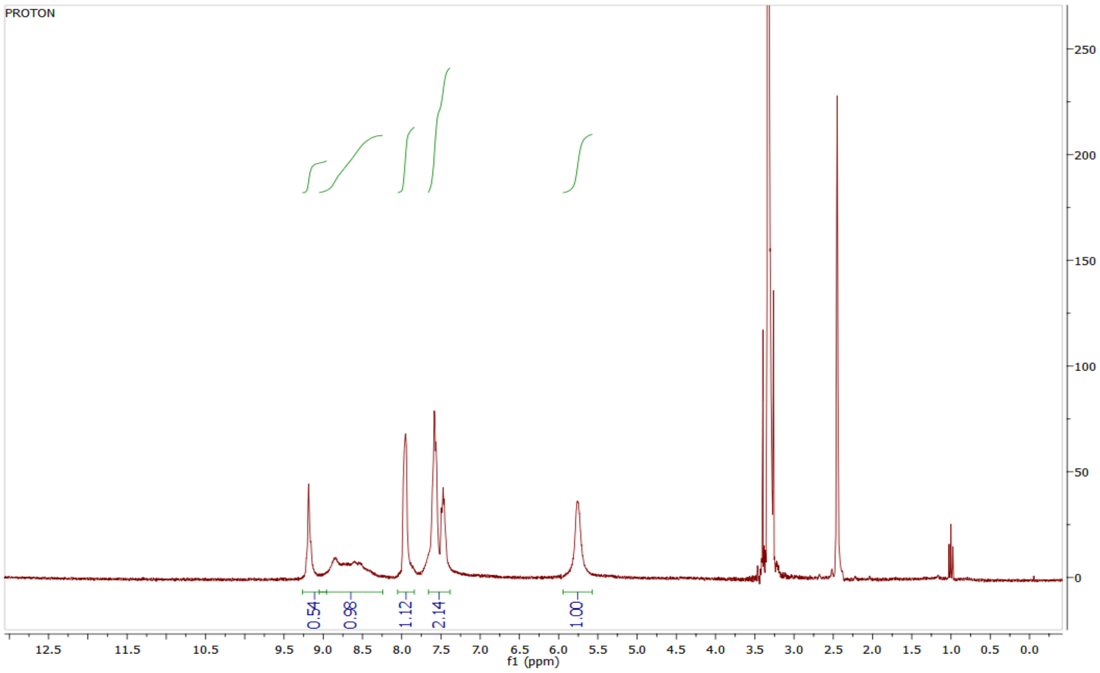

Figure S10. ^{1}H-NMR spectrum of compound **4** in DMSO-d_6.

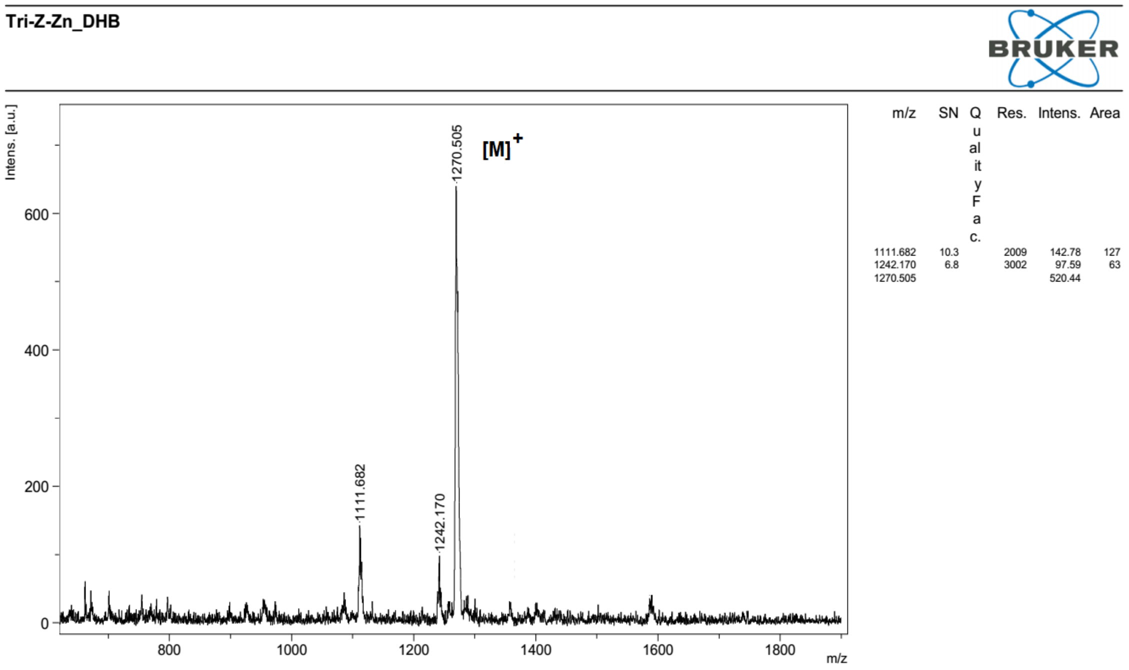

Figure S11. The MALDI-TOF mass spectrum of zinc complex **4**.

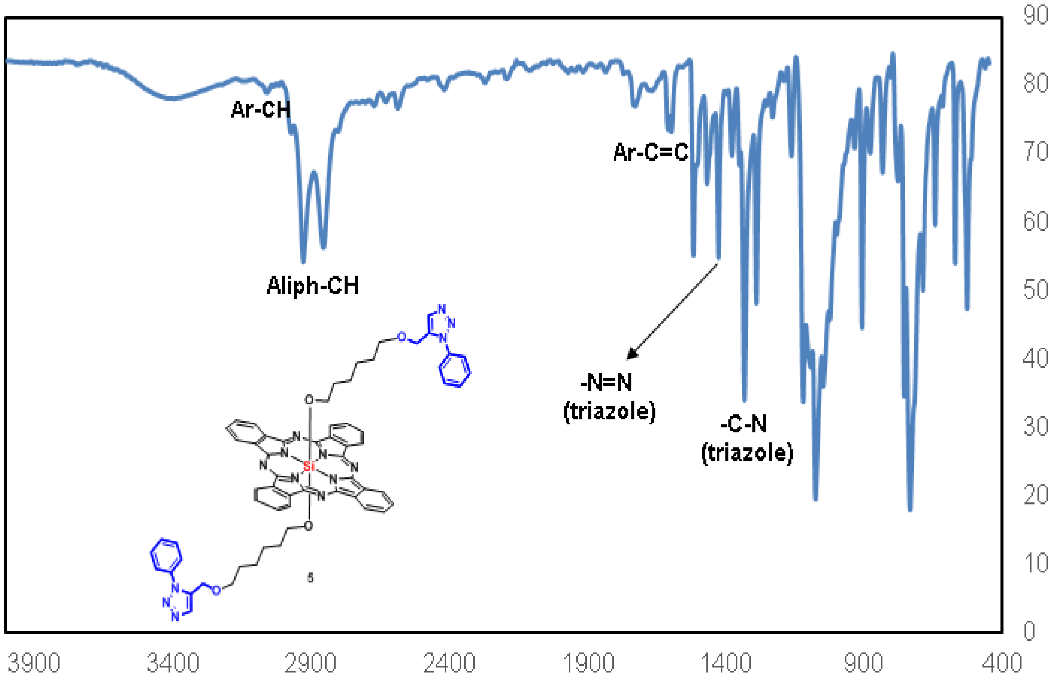

Figure S12. FT-IR spectrum of silicon complex **5**.

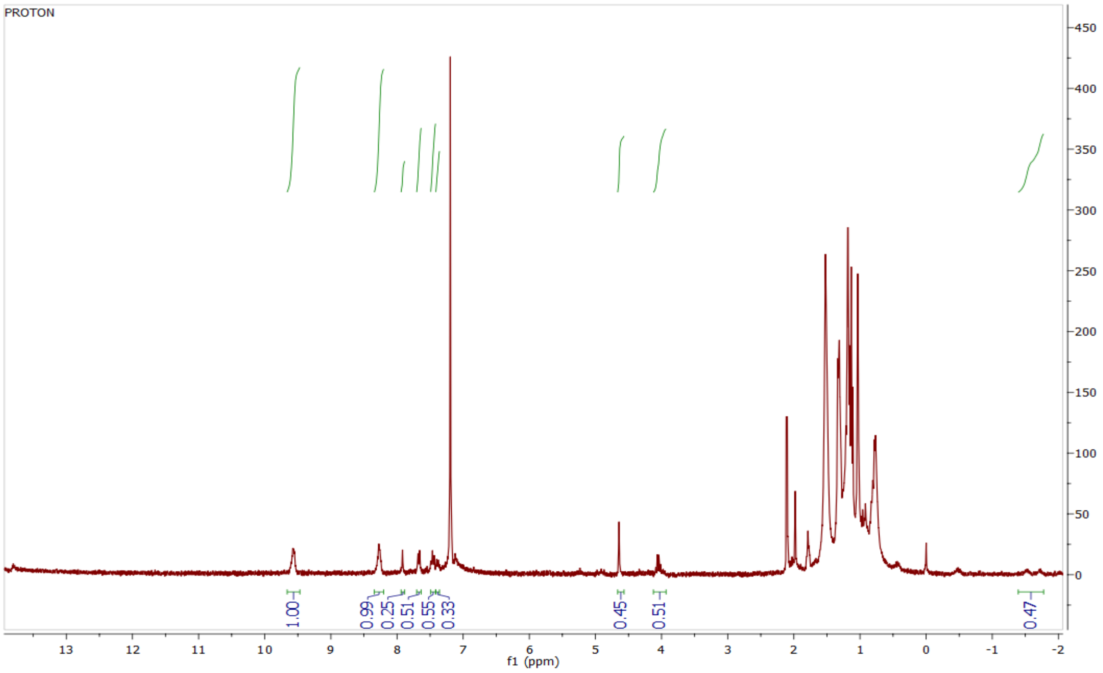

Figure S13. ^{1}H-NMR spectrum of compound **5** in $CDCl_3$.

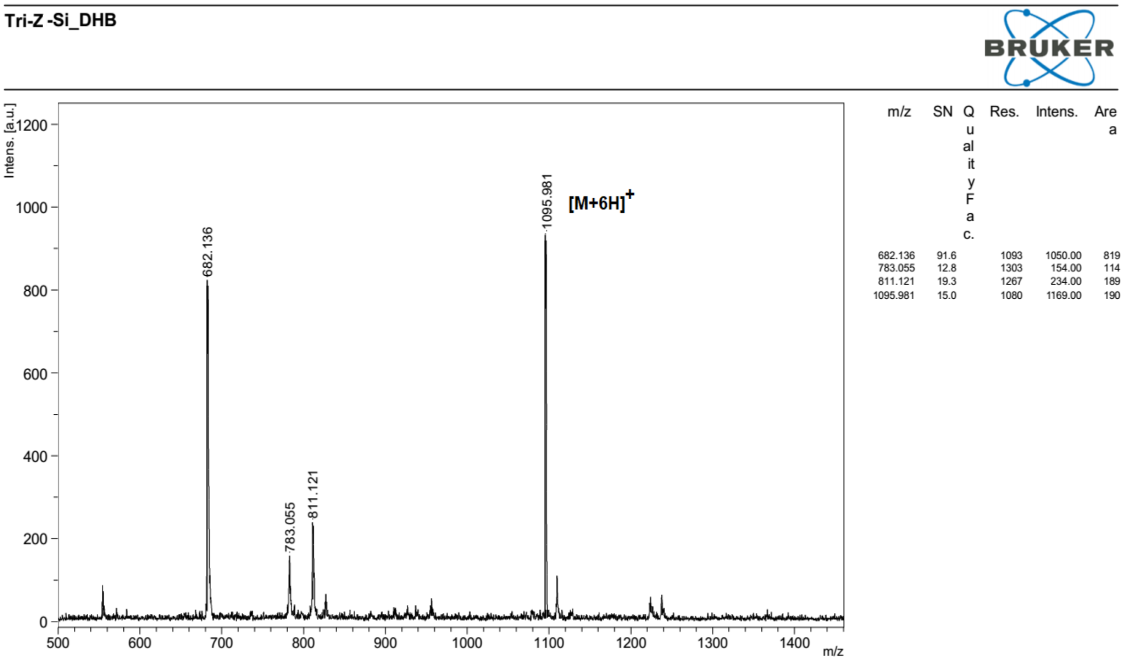

Figure S14. The mass spectrum of the complex **5**.

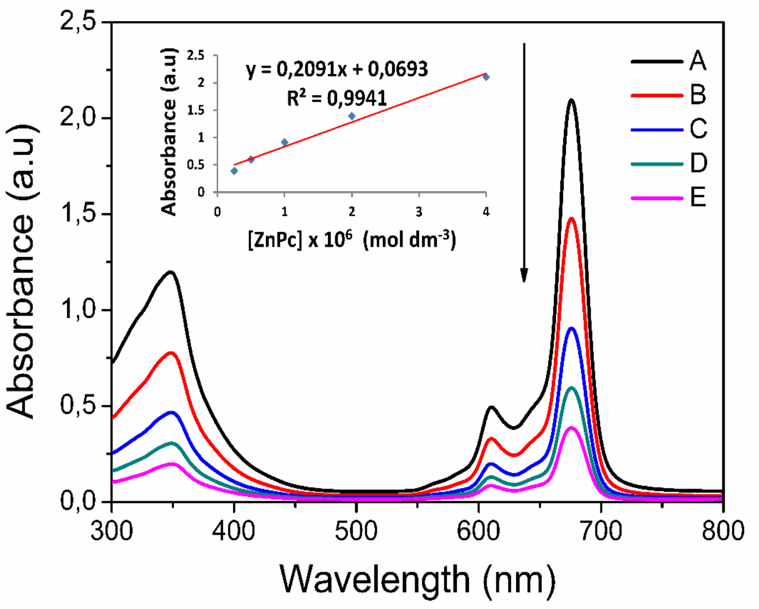

Figure S15. Electronic absorption spectra of complex **4** (ZnPc) in THF at different concentrations:(A) 40 x 10^{-6}, (B) 20 x 10^{-6}, (C) 10 x 10^{-6}, (D) 5 x 10^{-6}, (E) 2.5 x 10^{-6} mol dm^{-3}. The inset shows calibration plot for Q band maximum.

3. Photophysical and photochemical parameters

Fluorescence excitation and emission spectra were recorded on a Shimadzu spectrofluorometer using 1 cm path length cuvette at room temperature. Fluorescence quantum yields (Φ_F) are determined in DMSO by the comparative method using by equation 1.[3]

$$\Phi_F = \Phi_{F(Std)} \frac{F \, . \, A_{Std} . \, n^2}{F_{Std} . \, A \, . \, n^2_{Std}} \tag{1}$$

where F and F_S are the areas under the fluorescence emission curves of the samples (**4** and **5**) and the standard, respectively. A and A_{Std} are the respective absorbances of the samples and standard at the excitation wavelengths, respectively. n^2 and n^2_{Std} are the refractive indices of

solvents used for the sample and standard, respectively. Unsubstituted ZnPc (Φ_F = 0.20) [4] was employed as the standard in DMSO. The absorbance of the solutions at the excitation wavelength ranged between 0.04 and 0.05.

Singlet oxygen quantum yield (Φ_Δ) determinations were carried out by using the experimental set-up described in the literature.[5–7] Typically, a 3 mL portion of the respective unsubstituted zinc (II) phthalocyanine (ZnPc) and the studied phthalocyanine solutions containing the singlet oxygen quencher was irradiated in the Q band region with the photo-irradiation set-up described in references.[6–8] Singlet oxygen quantum yields (Φ_Δ) were determined in DMSO using the relative method with unsubstituted zinc (II) phthalocyanine (ZnPc) as a reference. DPBF was used as the chemical quencher for singlet oxygen in DMSO. Equation **2** was employed for the calculations:

$$\Phi_\Delta = \Phi_\Delta^{Std} \frac{R \, . \, I_{abs}^{Std}}{R^{Std} \, . \, I_{abs}} \qquad \textbf{(2)}$$

where Φ_Δ^{Std} is the singlet oxygen quantum yield for the standard unsubstituted zinc (II) phthalocyanine (ZnPc) (Φ_Δ^{Std}= 0.67 in DMSO). R and R^{Std} are the DPBF photobleaching rates in the presence of studied phthalocyanine compounds and standard, respectively. I_{abs} and I_{abs}^{Std} are the rates of light absorption by the studied phthalocyanine compounds and standard, respectively. To avoid chain reactions induced by DPBF in the presence of singlet oxygen, the concentration of quencher (DPBF) was lowered to ~$5x10^{-6}$ M. Solutions of sensitizers containing DPBF were prepared in the dark and irradiated in the Q band region using the photoirradiation setup. DPBF degradation at 417 nm was monitored. The light

intensity 7.15 x 10^{15} photons s^{-1} cm^{-2} was used for Φ_Δ determinations. The absorption band of DPBF reduced by light irradiation (Fig.8 and Fig S16). The zinc complex (**4**) (Φ_Δ =0.76) and silicon complex (**5**) (Φ_Δ =0.32) in DMSO was occurred singlet oxygen quantum yield.

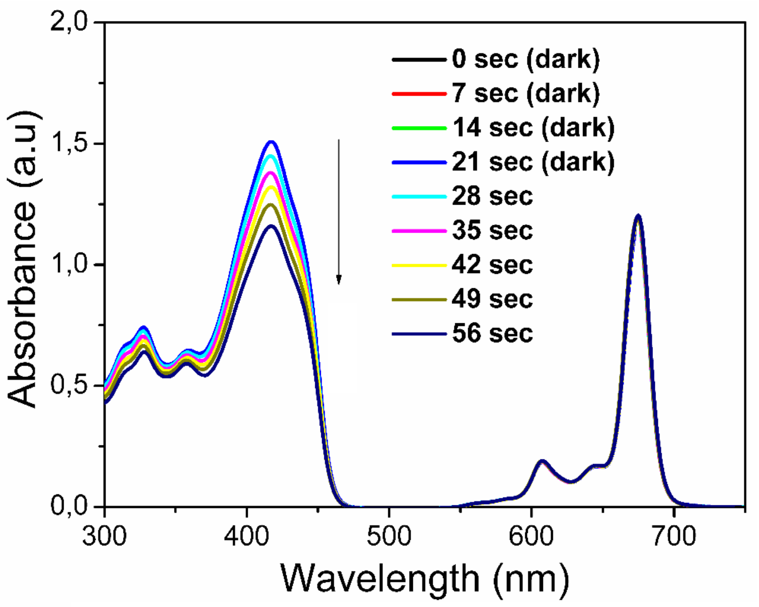

Figure S16. Reaction of the singlet oxygen generated by photo-sensitization with 1,3-diphenyl-isobenzofuran (DPBF) in dimethyl sulfoxide (DMSO) in the presence of silicon phthalocyanine complex (**5**). For the first 21 sec, the solution was kept in the dark; thereafter, it was irradiated with a light source (650 nm cut on filter) for 35 sec. The total volume was adjusted to 3.0 mL. Absorbance spectra were recorded in 7 sec intervals.

4. Electrochemical Parameters

Electrochemical studies were carried out with a PARSTAT 2273 potentiostat/galvanostat. Cyclic voltammograms (CVs) were recorded in degassed 0.1 M tetrabutylammonium tetrafluoroborate ($TBABF_4$)/DCM solution containing 0.5 mM dye at scan rate of 0.100 V s^{-1}. A glassy carbon electrode (GCE, 3 mm diameter), a Pt wire, and an Ag/AgCl wire were served as the working, counter and reference electrodes, respectively. The peak separation

potential (ΔE_p) of ferrocene/ferrocenium (Fc/Fc^+) redox couple as an external reference under our experimental conditions was 91 mV.

5. Photovoltaic Parameters

A double layer TiO2 photoanode was prepared by previously reported procedures[9], as described below. An adhesive tape (Scotch™, 3M) with a window of 0.196 cm^2 area and 25 μm depth was applied to the conductive surface of the fluorine-doped tin oxide-coated glass electrode (FTO) (Solaronix, TCO22−15, 15 Ω cm−2). A thin TiO_2 blocking layer was deposited onto FTO by dipping the tape-coated FTO in 40 mM aqueous solution of $TiCl_4$ for 30 min at 70 °C followed by rinsing with deionized water. Then, an anatase TiO_2 paste with an average particle size of ~ 20 nm (Solaronix Ti-Nanoxide T/SP) was coated on this electrode by the doctor blade method. The tape was removed and the TiO_2 electrode was gradually at 500 °C, followed by heating at the same temperature for 30 min in air. After cooling, the coating and heating processes were repeated over the previously coated electrode to obtain double layer TiO_2. The TiO_2 electrode was cooled to room temperature and again treated with the $TiCl_4$ solution, which was then heated at 500 °C for 30 min. After cooling to 80 °C, the resultant TiO_2 photoanode was sensitized with 0.3 mM phthalocyanine complex 4 and 5 in an ethanol/THF (v/v: 1/1) solution for 24 h. 1 mM chenodeoxycholic acid (CDCA) as a co-adsorbent was also added into the dye solution to suppress the aggregation of dye. As a reference DSSC, the TiO_2 photoanode was also dipped in an ethanol solution containing 0.5 mM N719 (Solaronix, Ruthenizer 535-bisTBA) and 5 mM CDCA for 6 h. Pt-coated FTO was used as counter electrode. The sensitized TiO_2 photoanode and the counter electrode were assembled using a 25 μm–thick sealing spacer (Solaronix, Meltonix 1170−25) and an I^-/I_3^- electrolyte solution (Solaronix, Iodolyte HI−30).

The DSSC's photocurrent–voltage (*J–V*) characteristics were carried out using the above-mentioned potentiostat/galvanostat under a solar simulator (96000, Newport, USA) with AM 1.5G illumination (100 mW cm^{-2} = 1 sun). The measurement of incident photon to current conversion efficiency (IPCE) was recorded by using a monochromator (74004, Oriel, USA). The cells were covered with a black mask (0.196 cm^2) during the photovoltaic measurements. For each dye, at least three DSSCs were fabricated and the averaged data were selected. The electrochemical impedance spectroscopy (EIS) measurements were performed by using the potentiostat/galvanostat at an applied bias voltage equivalent to the open-circuit voltage (V_{OC}) of each DSSC in the dark between 0.1 Hz and 1 MHz.

References

1 Perrin DD, Armarego WLF, *Purification of Laboratory Chemicals (2nd edn)*, Oxford, Pergamon P., 1989.

2 J. T. Simmons, J. R. Allen, D. R. Morris, R. J. Clark, C. W. Levenson, M. W. Davidson and L. Zhu, *Inorg. Chem.*, 2013, **52**, 5838–5850.

3 M. D. Maree, T. Nyokong, K. Suhling and D. Phillips, *J. Porphyr. Phthalocyanines*, 2002, **06**, 373–376.

4 A. Ogunsipe, J. Chen and T. Nyokong, *New J. Chem.*, 2004, **28**, 822–827.

5 A. Ogunsipe and T. Nyokong, *J. Photochem. Photobiol. A*, 2005, **173**, 211–220.

6 J. H. Brannon and D. Magde, *J. Am. Chem. Soc.*, 1980, **102**, 62–65.

7 I. Seotsanyana-Mokhosi, N. Kuznetsova and T. Nyokong, *J. Photochem. Photobiol. A Chem.*, 2001, **140**, 215–222.

8 A. Ogunsipe and T. Nyokong, *J. Photochem. Photobiol. A Chem.*, 2005, **173**, 211–220.

9 Y. Hu, S. Yellappa, M. B. Thomas, R. G. W. Jinadasa, A. Matus, M. Shulman, F. D'Souza and H. Wang, *Chem. - An Asian J.*, 2017, **12**, 2749–2762.
